# Gut microbiota depletion accelerates hematoma resolution and neurological recovery after intracerebral hemorrhage via p-coumaric acid-promoted Treg differentiation

**DOI:** 10.7150/thno.113764

**Published:** 2025-06-09

**Authors:** Yonghe Zheng, Yin Li, Xuchao He, Yuhan Zhu, Shiyu Xu, Yirong Feng, Yirui Kuang, Huaijun Chen, Linfeng Fan, Huaping Huang, Libin Hu, Xian Yu, Jianan Wu, Lingji Jin, Junwen Hu, Xiongjie Fu, Hanhai Zeng, Shenglong Cao, Lin Wang

**Affiliations:** 1 Department of Neurosurgery & Key Laboratory of Precise Treatment and Clinical Translational Research of Neurological Diseases, Second Affiliated Hospital, School of Medicine, Zhejiang University, Hangzhou, China.; 2 State Key Laboratory of Transvascular Implantation Devices, Hangzhou, China.

**Keywords:** Regulatory T cells, gut microbiota, p-coumaric acid, intracerebral hemorrhage, hematoma resolution.

## Abstract

Hematoma volume significantly influences the prognosis of patients with intracerebral hemorrhage (ICH). Effective resolution of hematoma through enhanced clearance mechanisms and reduced hematoma lysis is essential for neurological recovery following ICH. Regulatory T cells (Tregs), known for their anti-inflammatory properties, exert neuroprotective effects in various central nervous system disorders. Additionally, gut microbiota profoundly impacts Treg development through multiple regulatory pathways. Nonetheless, the precise roles of Tregs and gut microbiota in facilitating hematoma resolution after ICH remain unclear. This study, therefore, aimed to investigate the contributions of Tregs and gut microbiota to hematoma resolution post-ICH, as well as the underlying mechanisms.

**Methods:** The impact of gut microbiota depletion on neurological deficits and hematoma resolution, including erythrophagocytosis and erythrocyte lysis, was assessed using antibiotic cocktail (ABX) gavage administered prior to ICH induction in mice. Flow cytometry analysis and targeted cell depletion techniques were employed to identify peripheral immune cell populations mediating the beneficial effects of gut microbiota depletion on neurological recovery and hematoma resolution. The functional roles of Tregs in erythrophagocytosis, erythrocyte lysis, and associated downstream molecular signaling pathways were investigated through adoptive Treg transfer experiments. The mechanisms underlying Treg population expansion post-microbiota depletion in ICH mice were explored using multi-omics analysis of serum and fecal metabolites via mass spectrometry and fecal microbial composition using 16S rRNA sequencing. Additionally, the effects of p-coumaric acid (PCA) gavage and clindamycin-mediated depletion of PCA-metabolizing gut microbiota on Treg abundance, hematoma resolution, and neurological recovery post-ICH were assessed.

**Results:** Gut microbiota depletion by ABX gavage increased brain Treg populations, thereby enhancing erythrophagocytosis, suppressing erythrocyte lysis, and ultimately promoting hematoma resolution and neurological recovery. Adoptive Treg transfer experiments further established that Tregs facilitate scavenger pathway-mediated erythrophagocytosis and suppress complement-mediated erythrocyte lysis. These effects occurred via upregulation of efferocytosis receptors (MERTK and AXL), ligands (Gas6 and C1q), and the hemoglobin scavenger receptor CD163, alongside downregulation of complement C3 expression and reduced formation of membrane attack complexes (MACs). Multi-omics analysis demonstrated that ABX gavage eliminated PCA-metabolizing microbiota, thereby increasing PCA concentrations in serum and feces. Elevated PCA levels promoted peripheral Treg differentiation by inhibiting the PKCθ-AKT-FoxO1/3a signaling pathway, leading to higher brain Treg numbers. PCA gavage and clindamycin treatment similarly enhanced brain Treg populations, accelerated hematoma resolution, and improved neurological recovery following ICH.

**Conclusion:** Gut microbiota depletion facilitates hematoma resolution and neurological recovery through PCA-mediated induction of Treg differentiation.

## Introduction

Intracerebral hemorrhage (ICH) represents a severe stroke subtype characterized by high morbidity and mortality, occurring due to ruptured blood vessels and subsequent extravasation of blood into brain parenchymal tissue 1. Following vessel rupture, extravasated blood rapidly triggers coagulation cascades, leading to hematoma formation 2. Hematoma volume serves as an independent indicator of early disease severity and strongly predicts long-term neurological outcomes in patients with ICH. Rapid hematoma expansion exerts mechanical compression on brain tissue, contributing to primary brain injury 3. Subsequent hematoma lysis releases hemoglobin and its degradation products, furtherly exacerbating secondary brain injury 4. The MISTIE III trial demonstrated improved neurological outcomes in patients whose hematoma size was reduced to less than 15 mL, highlighting hematoma resolution as a critical therapeutic goal for patients with ICH 5.

Post-ICH hematoma resolution involves two key processes: hematoma lysis and hematoma clearance. The complement cascade is activated in the brain after ICH, leading to C3 cleavage and the formation of membrane attack complexes (MACs) 6. MAC-induced pore formation disrupts erythrocyte membrane permeability, ultimately causing erythrocyte lysis. This process releases erythrocyte-derived toxic products, significantly contributing to secondary brain injury post-ICH 7. Concurrently, an endogenous hematoma clearance system is activated, primarily involving microglia and macrophages that phagocytose erythrocytes and their degradation products 8. Erythrocyte efferocytosis and the hemoglobin-CD163 scavenger pathway represent essential mechanisms through which microglia and macrophages facilitate hematoma clearance 9, 10. Therapeutic strategies targeting enhanced scavenger pathway-mediated hematoma clearance and attenuated complement-mediated hematoma lysis thus offer potential avenues for promoting hematoma resolution and neurological recovery post-ICH.

Regulatory T cells (Tregs) constitute a minor subpopulation of CD4^+^ T cells characterized by the expression of signature markers, such as CD25 and the transcription factor forkhead box p3 (Foxp3) 11. As immunoregulatory cells, Tregs curtail excessive inflammatory responses and limit immune activation 12. Previous research has demonstrated beneficial roles of Tregs in cerebrovascular disorders; for example, Tregs attenuate blood-brain barrier permeability and confer neuroprotection during cerebral ischemia 13. Adoptive Treg therapy attenuates cerebral edema and mitigates brain injury following subarachnoid hemorrhage 14. Additionally, in experimental ICH models, Tregs promote M2 microglia/macrophage polarization, suppress inflammatory responses, and alleviate early brain damage 15. Nevertheless, the precise contribution of Tregs to hematoma resolution following ICH remains insufficiently understood.

Gut microbiota have been widely recognized for their critical roles in regulating Treg development. Compared to germ-free mice, specific pathogen-free (SPF) mice exhibit higher Treg populations across multiple tissues 16. Gut-derived bacteria signals modulate Treg differentiation through various mechanisms, including microbial metabolites 17, dendritic cell modulation 18, and bacterial cell-wall components 19. Gut microbiota-derived metabolites, in particular, are critical regulators of peripheral Treg (pTreg) differentiation. For example, butyrate, the short-chain fatty acid produced by gut microbiota, promotes Treg differentiation both *in vitro* and *in vivo* 20. Additionally, gut microbiota-derived linolenic acid, an essential polyunsaturated fatty acid, enhances Treg development and exerts anti-inflammatory effects in colitis models 21. These findings collectively suggest the significant involvement of gut microbiota and their metabolites in the induction and maturation of pTregs. Clinical evidence revealed that altered gut microbiota composition correlates with disease progression, severity, and clinical outcomes among patients with hypertensive ICH 22. Previously, preclinical studies demonstrated that gut microbiota dysbiosis triggered by ICH could be reversed through fecal microbiota transplantation (FMT), reducing infiltration of pro-inflammatory Th1 and Th17 T cells and subsequently alleviating neuroinflammation 23. However, the precise role of gut microbiota and their metabolites in modulating Treg-mediated hematoma resolution after ICH has yet to be fully elucidated.

In this study, the impact of gut microbiota on hematoma resolution and Treg cell expansion was investigated using an antibiotic cocktail (ABX) gavage model to deplete gut microbiota in mice prior to experimentally induced ICH. Gut microbiota depletion significantly enhanced erythrophagocytosis, inhibited erythrocyte lysis, and promoted hematoma resolution, leading to improved neurological recovery after ICH. The accelerated hematoma resolution following gut microbiota depletion was mediated by an expanded Treg population in the brain. Adoptive transfer experiments further confirmed that Tregs promoted hematoma resolution by upregulating efferocytosis-related receptors (MERTK and AXL), ligands (Gas6 and C1q), and the hemoglobin scavenger receptor CD163. Additionally, Tregs reduced complement component C3 expression and subsequent MAC formation, thereby facilitating erythrophagocytosis, suppressed erythrocyte lysis, and ultimately promoting hematoma resolution and neurological recovery. Multi-omics analysis integrating serum and fecal metabolite profiling through mass spectrometry with fecal 16S rRNA sequencing revealed that ABX gavage eliminated gut bacteria responsible for metabolizing p-coumaric acid (PCA), leading to increased serum and fecal PCA levels post-ICH. Elevated PCA levels facilitated naïve CD4^+^ T cells differentiation into Treg by inhibiting the protein kinase C-theta (PKCθ)-protein kinase B (AKT)-forkhead box O1/3a (FoxO1/3a) signaling pathway. Pharmacological administration of PCA and clindamycin-mediated depletion of PCA-metabolizing microbiota further enhanced the brain Treg population, accelerated hematoma resolution, and improved neurological recovery following ICH.

## Materials and Methods

### Ethics statement

All animal experiments were conducted in compliance with the ethical guidelines and regulations of the Ethics Committee of the Second Affiliated Hospital, Zhejiang University, China (approval no. AIRB-2022-082).

### Mice

Eight-week-old male C57BL/6J mice were obtained from Gem Pharmatech (Nanjing, China). and housed under specific pathogen-free (SPF) conditions with a 12-hour light/dark cycle, in accordance with standard operating procedures.

For gut microbiota depletion, mice received an ABX consisting of vancomycin (100 mg/kg), neomycin sulfate (200 mg/kg), metronidazole (200 mg/kg), and ampicillin (200 mg/kg) administered by oral gavage daily for 7 consecutive days. ABX treatment was discontinued 2 days prior to ICH induction to minimize potential off-target effects. Mice in the SPF control group received sterile drinking water by oral gavage during the same period.

For FMT, fresh fecal samples were collected from donor SPF mice between 9:00 and 10:00 AM and diluted in sterile phosphate-buffered saline (PBS) at a concentration of 120 mg/ml. Stool samples were incubated in cold PBS for 5 minutes, homogenized for 10 minutes, and further incubated at 4 °C for 10 minutes. Recipient ABX-treated mice received 200 µL of fecal supernatant or sterile PBS by oral gavage daily, starting 2 days prior to ICH induction and continuing until sacrifice.

For Treg depletion, 300 µg of CD25-specific monoclonal antibody (clone 16-0251-85, Invitrogen) or isotype control antibody (mouse IgG1, Invitrogen) was administered via intraperitoneal injection 48 hours before ICH induction.

For adoptive Treg transfer, CD4^+^CD25^+^ Tregs were isolated using a previously described method 24. Single-cell suspensions were prepared from mouse spleens using the gentleMACS Dissociator (Miltenyi Biotec, Germany). CD4^+^CD25^+^ Tregs were purified through magnetic separation using CD4-negative selection followed by CD25-positive selection with the Regulatory T Cell Isolation Kit (Miltenyi Biotec, Germany), according to the manufacturer's instructions. Recipient mice were intravenously injected with 2×10^6^ freshly enriched Tregs in 0.1 mL PBS via the tail vein, 30 minutes after ICH induction. Vehicle control mice received 100 µL of PBS via tail vein injection at the same time point.

For PCA and clindamycin treatments, mice received 400 µL of PCA (100 mg/kg) or clindamycin (67 mg/kg; Maokang, Shanghai) by oral gavage once daily for 3 consecutive days following ICH induction. The Vehicle group received 400 µL of PBS by oral gavage on the same schedule.

### Experimental design

Twelve independent experiments were conducted in this study. The overall experimental workflow is illustrated in Figure S5, and the sample size for each experiment is detailed in Table S1. Mice exhibiting intraventricular hemorrhage at the time of brain sample collection were excluded from analysis. To ensure experimental rigor, ICH modeling, drug administration, experiment tests and result recording were performed by different investigators blinded to group allocation.

**Experiment 1:** To investigate the effects of gut microbiota on hematoma resolution and neurological deficits after ICH, SPF mice received an ABX via oral gavage for 7 days to deplete gut microbiota, while control mice received vehicle (sterile drinking water) over the same period of time. ICH was induced via autologous blood injection in both groups. On days 3 and 7 post-ICH, neurological performance and hematoma volume were assessed. Fresh fecal samples were collected on day 3 for 16S rRNA gene sequencing to evaluate microbial composition. To explore the impact of gut microbiota on peripheral infiltrating cells, flow cytometry was performed on brain tissue from the lesioned hemisphere on day 3 following ICH.

**Experiment 2**: To further determine the role of gut microbiota following ICH, FMT was performed to restore microbial composition in ABX-treated mice. Recipient ABX mice received 200 µL of either fecal supernatant from donor SPF mice or vehicle (PBS) by oral gavage once daily, starting 2 days prior to ICH induction and continuing until sacrifice. ICH was induced by injecting autologous blood into the striatum in SPF, ABX, and FMT groups. Neurological tests and magnetic resonance imaging were performed on day 3 following ICH. On day 3 post-ICH, brain samples were collected for hematoma volume measurement. Fluorescent-labeled blood was injected into the striatum to induce the ICH model. Erythrophagocytosis in the perihematomal region was assessed by flow cytometry on 3 days after ICH induction. To further confirm the impact of gut microbiota on brain Treg populations, brain tissue from the lesioned hemisphere was collected for flow cytometry in SPF, ABX, and FMT groups on day 3 following ICH.

**Experiment 3:** To confirm the role of Treg cells in ABX-mediated hematoma resolution, ABX-treated mice received intraperitoneal injections of anti-CD25 monoclonal antibody or isotype control (IgG) 48 hours before ICH induction. Neurological tests, hematoma volume measurement, erythrophagocytosis assays, and MRI were performed on day 3 following ICH in SPF + IgG, ABX + IgG, and ABX + anti-CD25 groups.

**Experiment 4:** To further determine the role of Tregs in hematoma resolution after ICH, adoptive Treg transfer was performed. ICH was induced by injection of autologous blood. Mice received Treg or vehicle (PBS) via tail vein injection 30 minutes after ICH induction. Neurological tests and MRI were conducted in Sham + Vehicle, ICH + Vehicle, and ICH + Treg groups on day 3 following ICH. On day 3, brain samples were collected for hematoma volume measurement, enzyme-linked immunosorbent assay, and western blotting. Fluorescent-labeled blood was injected into the striatum to induce the ICH model. Erythrophagocytosis in the perihematomal region was assessed by flow cytometry in Sham + Vehicle, ICH + Vehicle, and ICH + Treg groups on day 3 following ICH.

**Experiment 5:** To determine the underlying mechanism of brain Treg population enhancement in ABX mice after ICH, ICH was induced by injection of autologous blood. On day 3 following ICH, the brain, gut, spleen, thymus, and blood were collected from SPF, ABX, and FMT groups. Flow cytometry was performed to evaluate Treg populations and their proliferation in these tissues.

**Experiment 6:** To assess the effect of gut microbiota on Treg differentiation, naïve CD4^+^ T cells were magnetically sorted and treated with serum collected on day 3 following surgery from SPF + Sham mice, SPF + ICH mice, ABX + Sham mice, and ABX + ICH mice. On day 3 following serum administration, cells were collected for flow cytometry to evaluate Treg differentiation.

**Experiment 7:** To identify serum factors responsible for triggering naïve CD4^+^ T cell differentiation into Treg, serum samples were collected on day 3 following surgery from SPF + Sham, ABX + Sham, SPF + ICH, and ABX + ICH groups for metabolomic analysis. The level of PCA in serum and feces was measured in SPF + Sham, SPF + ICH, and ABX + ICH groups on day 3 following ICH.

**Experiment 8:** To determine which specific serum metabolite was responsible for inducing Treg differentiation, naïve CD4^+^ T cells were magnetically sorted and treated with Tauro-β-muricholic acid (TβMCA), PCA, taurocholic acid (TCA), or vehicle (0.1% dimethyl sulfoxide solution). On day 3 following metabolite treatment, cells were collected for flow cytometry to detect Treg differentiation.

**Experiments 9 and 10:** To investigate the molecular mechanism by which PCA induces naïve CD4^+^ T cell differentiation into Treg, naïve CD4^+^ T cells were magnetically sorted and treated with PCA or vehicle (0.1% dimethyl sulfoxide solution). Cells were collected at 0 hours, 36 hours, and 72 hours after treatment for western blot analysis. In a parallel experiment, naïve CD4^+^ T cells were treated with vehicle (0.1% dimethyl sulfoxide solution), PCA or PCA combined with phorbol-12-myristate-13-acetate (PMA), and flow cytometry was performed to evaluate Treg differentiation.

**Experiment 11:** To assess the impact of PCA on brain Treg populations, hematoma resolution, and neurological recovery after ICH *in vivo*, ICH was induced by injection of autologous blood. Mice received oral gavage of PCA (100 mg/kg) or an equal volume of vehicle (PBS) once daily for 3 consecutive days after ICH induction. Neurological tests were conducted in ICH + Vehicle and ICH + PCA groups on day 3 post-ICH. On the same day, brain tissue was collected for hematoma volume measurement and flow cytometry analysis.

**Experiment 12:** To investigate the effects of PCA-metabolizing microbiota depletion on the brain Treg population, hematoma resolution, and neurological recovery, ICH was induced by injection of autologous blood. Mice received oral gavage of clindamycin (67 mg/kg) or an equal volume of vehicle (PBS) once daily for 3 consecutive days after ICH induction. Neurological tests were conducted in ICH + Vehicle and ICH + clindamycin groups on day 3 following ICH. On the same day, brain tissue was collected for hematoma volume measurement and flow cytometry analysis.

### ICH model

The ICH model was established by injecting autologous blood into eight-week-old male C57BL/6J mice (25-30 g), as previously described 25. Anesthesia was induced by intraperitoneal injection of 1% pentobarbital sodium (40 mg/kg). Pain response during the surgical procedure was monitored using the toe pinch reflex. Thirty microliters of autologous blood were collected from the tail artery and injected into the ipsilateral striatum over 10 minutes at the following coordinates: 2.5 mm lateral and 3.0 mm deep at a 5° angle relative to bregma. Core body temperature was maintained at 37 °C throughout the procedure. Sham-operated mice underwent the same procedure, including needle insertion, but without blood injection.

### Gut microbiome sequencing

16S rRNA gene sequencing was performed by Cosmos Wisdom Biotech Co., Ltd. (Hangzhou, China) to analyze gut microbiota composition. On day 3 after ICH, fresh fecal samples were collected through abdominal massage to prevent contamination from exogenous bacteria. Genomic DNA was extracted using the PF Mag-Bind Stool DNA Kit (Omega Bio-tek, USA), in accordance with the manufacturer's instructions. Primers targeting conserved regions of the 16S rRNA gene, and sequencing adapters were added to the primer ends. PCR amplification was followed by product purification, quantification, and homogenization to construct sequencing libraries. Quality control was conducted using 1.0% agarose gel electrophoresis and a NanoDrop® ND-2000 spectrophotometer (Thermo Scientific Inc., USA). Sequencing was then performed using the Illumina NovaSeq 6000 platform. Reads were assembled, filtered, and clustered or denoised, followed by species annotation and relative abundance analysis. Alpha diversity, beta diversity, taxonomic composition, correlation analysis, and functional prediction were carried out to assess microbial community differences among samples.

### Behavioral testing

All behavioral assessments were conducted between 7:00 PM and 9:00 PM to maintain consistency across experimental groups.

Cylinder test 26: To assess forelimb use asymmetry, each mouse was placed in a transparent plastic cylinder and allowed to initiate a weight-shifting movement by freely rearing on day 3 or 7 following ICH. During each rear, the initial forelimb contacts with the cylinder wall (right, left, or both) was recorded until 20 rears were completed. The laterality index was calculated as (right - left) / 20, where higher positive values indicating preferential use of the right forelimb and more severe left hemiparesis.

Corner turn test 27: To evaluate motor coordination and sensorimotor integration, mice were allowed to enter a 30° transparent corner and choose a direction (left or right) to exit on day 3 or 7 after ICH. Ten trials were performed, and the direction preference was expressed as the percentage of right turns out of 10 trials.

Forelimb placement test 25: To assess vibrissae-evoked forelimb placing reflect, mice were held gently around the abdomen and their vibrissae were brushed against the edge of a countertop on day 3 or 7 after ICH. Each side was tested 10 times. Healthy control mice quickly placed the ipsilateral forelimb in response to vibrissae stimulation, whereas ICH mice typically exhibited a reduced or absent response. Injury severity was quantified as the number of successful forelimb placements out of 10 trials for each side.

### Residual hematoma volume measurement

Mice were sacrificed and transcardially perfused with PBS on day 3 or 7 after ICH. Coronal brain sections (1 mm thick) spanning the hematoma region were as previously described 28. Residual hematoma volume in digital images of brain slices was analyzed using ImageJ software (National Institutes of Health, USA). Residual hematoma volume, expressed in cubic millimeters, was calculated by multiplying the hematoma area in each section by the section thickness (1 mm).

### *In vivo* erythrophagocytosis

Murine blood was obtained via cardiac puncture. Erythrocytes were isolated using Ficoll (Sigma-Aldrich) gradient centrifugation and washed twice in PBS. Fluorescently labeled erythrocytes were prepared using the lipophilic fluorescent probe DiD (Biyuntian, Shanghai), in accordance with the manufacturer's instructions. Fluorescence-labeled erythrocytes were resuspended in autologous plasma at a 1:4 ratio, as previously described 9. Thirty microliters of this recombined fluorescent blood were injected into the striatum to induce the ICH model. Erythrophagocytosis in the perihematomal region was assessed by flow cytometry on day 3 post-ICH. The LIVE/DEAD^-^CD45^int/hi^CD11b^+^-DiD^+^ population was defined as erythrophagocytic microglia/macrophages.

### Magnetic resonance imaging (MRI)

Mice were anesthetized using a 1-3% isoflurane/air mixture, and core body temperature was maintained with a forced-air heating system. T2- and T2*-weighted MRI was performed on day 3 after ICH using a 7-T horizontal bore scanner (BioSpec 70/16 USR, Bruker BioSpin MRI, Ettlingen, Germany) equipped with a Bruker quadrature birdcage volume coil (T20061V3). Parameters for T2-weighted imaging included: field of view = 25 × 25 mm, slice thickness = 0.55 mm, matrix = 256 × 256, repetition time = 2500 ms, and effective echo time (TE_eff_) = 35 ms. Parameters for T2*-weighted imaging included: field of view = 25 × 25 mm, slice thickness = 0.55 mm, matrix = 256 × 256, repetition time = 1200 ms, echo time = 32 ms, and flip angle = 50°.

The T2* lesion was defined as a hypointense signal area and considered to represent the hematoma region. A hyperintense or isointense signal within the hematoma core was classified as a T2* hyperintense or isointense lesion. The lesion volume was calculated by summing the total hyperintense or isointense signal areas across all slices and multiplying by the section thickness. Ipsilateral ventricle compression, indicative of brain swelling, was assessed on every third coronal section centered on the anterior commissure layer in T2-weighted images. Swelling was quantified using the formula:

(ipsilateral hemisphere volume - contralateral hemisphere volume)/contralateral hemisphere volume × 100%

All measurements were repeated three times using ImageJ software and analyzed by a blinded investigator.

### Flow cytometry

Single-cell suspensions from brain tissue were prepared by harvesting the lesioned hemisphere on day 3 after ICH. Tissue was minced and homogenized using a tissue dissociation tube, followed by centrifugation at 900 × g for 20 minutes under minimal acceleration and deceleration in 30% Percoll to remove myelin debris. Erythrocytes were lysed using erythrocyte lysis buffer (2384218, Invitrogen), yielding a single-cell suspension.

For isolation of cells from the intestinal lamina propria, small and large intestines from the experimental mice were collected on day 3 after ICH. Peyer's patches, mesenteric fat, and luminal contents were removed, and intestines were cut into 1-cm segments. Tissue segments were incubated in 5 mL of Hank's balanced salt solution (HBSS) containing 10 mM HEPES, 5% fetal bovine serum (FBS), 5 mM ethylenediaminetetraacetic acid (EDTA), and 1 mM dithiothreitol at 37 °C in a shaking incubator at 150 rpm for 50 minutes. The resulting tissue suspension was vortexed for 20 seconds and filtered through a 70-μm cell strainer. Residual tissue fragments were washed with Ca^2+^/Mg^2+^-free PBS to remove EDTA, finely minced, and digested in 5 mL of HBSS containing 10 mM HEPES, 5% FBS, and 1 mg/mL collagenase IV (Invitrogen) at 37 °C in a shaking incubator at 150 rpm for 60 minutes. The digested suspension was filtered through a 40-μm nylon cell strainer and washed with 10 mL PBS. Single-cell suspensions from the intestinal lamina propria were obtained by centrifugation at 800 × g for 5 minutes at 4 °C.

Single-cell suspensions from the spleen, thymus, and peripheral blood were prepared on day 3 after ICH. Spleens and thymuses were isolated and placed on premoistened 70-μm cell strainers and gently dissociated using a 2-mL syringe plunger. Splenocytes were treated with 1 mL of erythrocyte lysis buffer for 5 minutes at room temperature and washed with PBS. Whole blood was collected via cardiac puncture into sodium heparin-coated tubes, followed by treatment with erythrocyte lysis buffer for 5 minutes at room temperature and washed with PBS. All suspensions were centrifuged at 600 × g for 5 minutes at 4 °C.

Each single-cell suspension was first incubated with an anti-CD16/CD32 antibody to block Fc receptors and reduce nonspecific binding. Cell viability was assessed using the Zombie NIR™ Fixable Viability Kit (423106, BioLegend, USA), following the manufacturer's protocol. Surface staining was performed by adding fluorochrome-conjugated antibodies at a 1:200 dilution, and incubating the samples in the dark at 4 °C for 30 minutes. The following antibodies were used: CD45 (phycoerythrin [PE], 12-0451-82, Invitrogen; AF700, 56-0454-82, Invitrogen), CD11b (phycoerythrin-cyanine 7 [PE-Cy7], 250112-82, Invitrogen), Ly6G (peridinin-chlorophyll-protein [PerCP]-Cy5.5, 127616, BioLegend), CD4 (allophycocyanin [APC], 17-0042-82, Invitrogen), and CD25 (Alexa Fluor 488, 53-0251-82, Invitrogen). For intracellular staining of nuclear markers, including Foxp3 and Ki67, cells were fixed and permeabilized using the Foxp3/Transcription Factor Fixation/Permeabilization Kit (00-5521, Invitrogen), according to the manufacturer's instructions. The permeabilized cells were then incubated with anti-FOXP3 antibody (eFluor 450, 48-5773-82, Invitrogen; PE, 126404, BioLegend) and anti-Ki67 antibody (PE-Cy7, 652426, BioLegend) under light-protected conditions at 4 °C. Samples were acquired using a flow cytometer (Beckman Coulter, USA), and data were analyzed using FlowJo software.

### Western blotting

Protein extraction was performed on brain tissue collected on day 3 after ICH and on cultured cells collected at 0, 36, and 72 hours following treatment. Tissue surrounding the hematoma and cultured cells were lysed in ice-cold radioimmunoprecipitation assay (RIPA) buffer supplemented with phosphatase and protease inhibitors (A32961, Invitrogen). Protein concentrations were determined, and equal amounts of total protein were subjected to sodium dodecyl sulfate-polyacrylamide gel electrophoresis (SDS-PAGE), followed by electrophoretic transfer onto polyvinylidene difluoride (PVDF) membranes at a constant current of 250 mV for 120 minutes. Membranes were blocked with 5% non-fat milk in Tris-buffered saline with 0.1% Tween 20 (TBST) for 1 hour at room temperature and incubated overnight at 4 °C with the following primary antibodies: anti-C1q (1:500, ab71940, Abcam), anti-C3 (1:2000, ab200999, Abcam), anti-AXL (1:500, bs-5180R, Bioss), anti-MERTK (1:1000, ab184086, Abcam), anti-Gas6 (1:500, bs-7549R, Bioss), anti-CD163 (1:1000, ab182422, Abcam), anti-p-PKCθ (1:1000, 9377T, Cell Signaling Technology), anti-PKCθ (1:1000, 13643, Cell Signaling Technology), anti-p-AKT (1:1000, 13038S, Cell Signaling Technology), anti-AKT (1:1000, 4691S, Cell Signaling Technology), anti-p-FoxO1 (1:1000, 9461T, Cell Signaling Technology), anti-FoxO1 (1:1000, 2880S, Cell Signaling Technology), anti-p-FoxO3a (1:500, AP0684, ABclonal), anti-FoxO3a (1:1000, A9270, ABclonal), and anti-β-actin (1:10000, Proteintech), which served as the internal loading control. After three washes with TBST, membranes were incubated with horseradish peroxidase-conjugated secondary antibodies (1:10000) for 1 hour at room temperature. Protein bands were visualized using a chemiluminescent substrate, and signal intensity was quantified using ImageJ software based on grayscale densitometry.

### Enzyme-linked immunosorbent assay (ELISA)

To quantify membrane attack complex (MAC) formation, brains were frozen *in situ* on day 3 after ICH. Hematoma tissue samples were collected and homogenized as previously described 7. MAC levels were quantified using a Mouse C5b-9 Terminal Complement Complex ELISA Kit (D721142, Sangong, Shanghai), in accordance with the manufacturer's instructions.

### Naïve CD4^+^ T-cell isolation and *in vitro* differentiation of Treg cell

Isolation of naïve CD4^+^ T cells from mouse spleens and *in vitro* differentiation into Treg cells were performed as previously described 18. Naïve CD4^+^ T cells were magnetically enriched from splenic lymphocytes using the MagniSort Mouse CD4^+^ Naïve T Cell Enrichment Kit (8804-6824, Invitrogen) in combination with MS columns (130-042-201, Miltenyi). The purity of the isolated naïve CD4^+^ T cell population (CD45^+^CD4^+^CD25^-^CD44^low^CD69L^high^) was confirmed to be 91.3% (Figure S1). Naïve CD4^+^ T cells were cultured in RPMI 1640 medium supplemented with 10% FBS and 1% penicillin-streptomycin. For Treg differentiation, the medium was further supplemented with 0.1 ng/mL transforming growth factor (TGF)-β1, 10 ng/mL interleukin (IL)-2, and CD3/CD28 T-activator DynaBeads (Invitrogen) at a 1:1 cell-to-bead ratio. For experiments involving serum exposure, 10 μL of serum was added to the culture medium. For metabolite treatment, cells were exposed to 100 μmol/L of Tauro-β-muricholic acid (TβMCA), PCA, or taurocholic acid (TCA), or to 5 ng/mL phorbol-12-myristate-13-acetate (PMA). A 0.1% dimethyl sulfoxide (DMSO) solution served as the vehicle control. After 3 days of culture, cells were collected, and the proportions of Treg cell population were analyzed by flow cytometry.

### Metagenomic sequencing

On day 3 following ICH, mice were anesthetized and blood was collected via cardiac puncture from the left ventricle into centrifuge tubes. Samples were allowed to coagulate at room temperature for 1 hour, followed by centrifugation at 1000 × g for 10 minutes. Serum supernatants were collected and stored at -80 °C until analysis. Targeted metabolomics profiling of mouse serum was conducted by Cosmos Wisdom Biotech Co., Ltd. (Hangzhou, China) using ultra-high performance liquid chromatography coupled with tandem mass spectrometry (UHPLC-MS/MS) to quantify functional small-molecule metabolites. Sample preparation and instrument parameter settings were conducted according to a previously published protocol 29. For statistical analysis, both principal component analysis and orthogonal projections to latent structures discriminant analysis (OPLS-DA) were applied. A V-plot derived from the OPLS-DA model was used to visualize the distribution and contribution of individual metabolites. Candidate biomarkers were identified using both multivariate and univariate statistical analyses, with the following selection criteria: variable importance in projection score > 1, *p*-value < 0.05, and |log_2_(fold change)| > 2. Metabolic pathway enrichment analysis was conducted using the iMAP platform (Cosmos Wisdom Biotech Co., Ltd.), and pathways with *p*-values < 0.05 were considered significantly altered and designated as candidate target pathways.

### Statistical analysis

Continuous data were expressed as mean ± standard deviation (SD) or median ±interquartile range (IQR), depending on data distribution and homogeneity of variance. For normally distributed data with equal variance, one-way analysis of variance (ANOVA) was utilized to compare differences among three or more groups, while unpaired t-tests were performed to compare differences between two groups. For normally distributed data with unequal variance, Brown-Forsythe tests and Welch's ANOVA were applied for multiple-group comparisons, and Welch's t-test was used for comparisons between two groups. For non-normally distributed data, the Kruskal-Wallis test was used to compare three or more groups, whereas the Mann-Whitney U test was utilized for two-group comparisons. For time-series data, two-way ANOVA followed by Tukey's multiple comparison test was carried out when the data exhibited normal distribution with equal variance; the Scheirer-Ray-Hare test was used for non-normally distributed time-series data. A *p*-value of < 0.05 was considered statistically significant. All statistical analyses were performed with GraphPad Prism 8.0 (GraphPad Prism, USA) and SPSS 23 (IBM Corp., USA).

## Results

### Gut microbiota depletion accelerates hematoma resolution and neurological recovery by enhancing erythrocyte phagocytosis and inhibiting hematoma lysis

**Figure 1:**
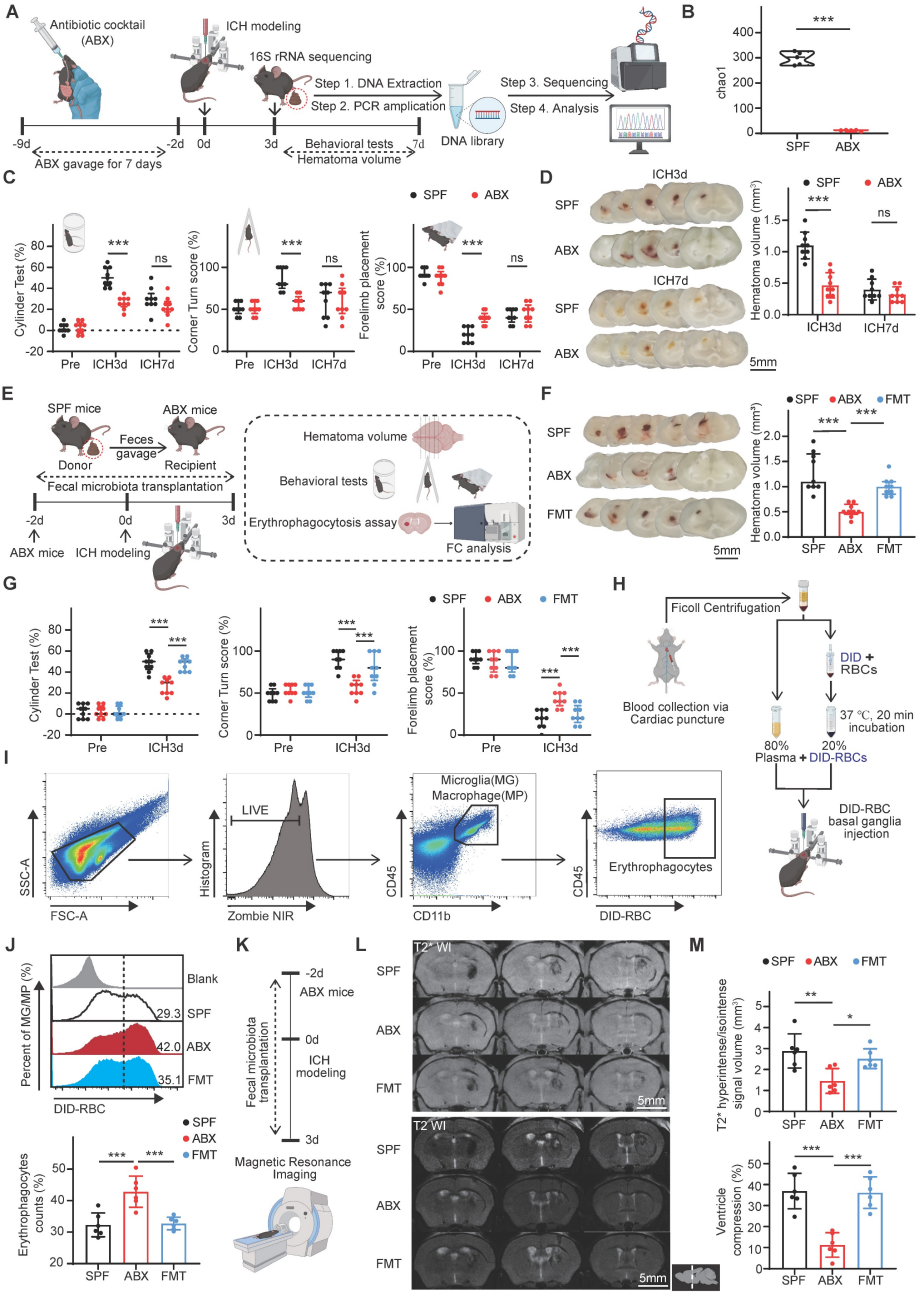
** Gut microbiota depletion promotes hematoma resolution and neurological recovery by enhancing erythrophagocytosis and inhibiting hematoma lysis on day 3 post-ICH.** (A) Schematic diagram of the experimental workflow illustrating the effects of antibiotic cocktail (ABX) gavage on gut microbiota composition, hematoma resolution, and neurological recovery after ICH in mice. Mice received ABX by oral gavage for 7 days, followed by a 2-day washout period, and subsequently underwent injection to induce ICH. Fecal samples were collected on day 3 post-ICH for 16S rRNA sequencing. Neurological function and residual hematoma volume were assessed on days 3 and 7 post-ICH. (B) Chao1 α-diversity indices of fecal microbiota in SPF and ABX-treated mice. Data are presented as median ± IQR (n = 5 per group). (C) Neurological function assessments in SPF and ABX-treated mice using the cylinder, corner, and forelimb placement tests. Data are presented as median ± IQR (n = 9 per group). (D) Representative coronal brain sections showing residual hematoma on days 3 and 7 post-ICH. Scale bar = 5 mm. Quantification of residual hematoma volume in SPF and ABX groups on days 3 and 7 post-ICH. Data are presented as mean ± SD (n = 9 per group). (E) Schematic diagram illustrating the effects of fecal microbiota transplantation (FMT) on hematoma resolution, neurological recovery, and erythrophagocytosis in ABX-treated mice post-ICH. (F) Representative coronal brain sections showing residual hematoma in SPF, ABX, and FMT groups on day 3 post-ICH. Scale bar = 5 mm. Quantification of residual hematoma volume in each group. Data are presented as median ± IQR (n = 9 per group). (G) Neurological function assessments in SPF, ABX, and FMT groups using the cylinder, corner, and forelimb placement tests on day 3 post-ICH. Data are presented as median ± IQR (n = 9 per group). (H, I) Schematic diagram illustrating the workflow for *in vivo* erythrophagocytosis analysis. Red blood cells (RBCs) were isolated via Ficoll gradient centrifugation from whole blood collected by cardiac puncture, labeled with the fluorescent dye DiD, and resuspended in autologous plasma (1:4 ratio). A 30-μL mixture was injected into the striatum to induce ICH. Microglia/macrophages engaging in erythrophagocytosis were identified by flow cytometry using the gating strategy for LIVE/DEAD^-^CD45^int/hi^CD11b^+^-DiD^+^ cells. (J) Percentages of erythrophagocytic microglia/macrophages in SPF, ABX, and FMT groups on day 3 post-ICH (n = 6 per group). Data are presented as mean ± SD. (K) Schematic diagram illustrating the experimental workflow used to evaluate the effects of FMT on hematoma lysis and ventricular compression in ABX-treated mice after ICH. Mice in each group were anesthetized on day 3 post-ICH, and T2- and T2*-weighted magnetic resonance imaging (MRI) was performed to assess hematoma lysis and ventricular compression. (L) Representative T2- and T2*-weighted MRI scans of SPF, ABX, and FMT groups on day 3 post-ICH. Dashed lines indicate hematoma location in the brain. (M) Quantification of T2* hyperintense or isointense lesion volume, and ipsilateral ventricular compression as an indicator of brain swelling (n = 6 per group). Data are presented as mean ± SD. Statistical significance: *p < 0.05, **p < 0.01, ***p < 0.001; ns = not significant.

To investigate the role of gut microbiota in hematoma resolution and neurological recovery following ICH, SPF mice were administered an ABX via oral gavage for 1 week prior to ICH induction to eliminate gut microbiota (Figure 1A). To confirm the effectiveness of gut microbiota depletion, fecal samples were collected from SPF and ABX mice on day 3 post-ICH and analyzed via 16S rRNA gene sequencing. α-diversity analysis showed a complete reduction of gut microbiota to zero in the ABX group, indicating successful elimination of gut microbiota by day 3 post-ICH (Figure 1B). Linear discriminant analysis effect size (LEfSe) investigation revealed significantly increased relative abundance of *Lactobacillus*, *Clostridiales*, *Desulfovibrio*, and *Muribaculaceae* in the gut microbiota of SPF mice compared with ABX mice. At the genus level, gut microbiota composition in SPF mice primarily consisted of *Ligilactobacillus*, *Muribaculaceae*, *Desulfovibrio*, *Clostridia*_UCG-014, and *Lactobacillus*, whereas the gut microbiota in ABX mice was dominated by *Enterococcus*, *Escherichia*-*Shigella*, and *Proteus* (Figure S2A, B). Neurological function and hematoma volume were then assessed in both groups. All mice exhibited comparable baseline neurological function prior to ICH. On day 3 post-ICH, ABX-treated mice demonstrated significantly improved neurological performance relative to SPF controls, although this difference was no longer observed by day 7 post-ICH (Figure 1C). Similarly, residual hematoma volume was significantly smaller in ABX mice on day 3 post-ICH, but had normalized to levels comparable with SPF mice by day 7 (Figure 1D). These findings suggest that gut microbiota depletion accelerates hematoma resolution and promotes transient neurological recovery post-ICH.

To further evaluate the role of gut microbiota, FMT was performed to restore microbial composition in ABX mice, followed by assessment of behavioral performance and hematoma volume in SPX, ABX, and FMT groups (Figure 1E). FMT reversed the neurological improvements and enhanced hematoma resolution observed in ABX mice (Figure 1F, G). These findings indicate that restoring the ICH-associated microbiota exacerbates neurobehavioral recovery and delays hematoma resolution.

Post-ICH hematoma resolution is driven by two primary factors: the rate of erythrocyte lysis and the extent of erythrophagocytosis by microglia/macrophages 30. To explore the impacts of gut microbiota on these processes, erythrophagocytosis and erythrocyte lysis were assessed. *In vivo* erythrophagocytosis was evaluated by injecting fluorescence-labeled autologous erythrocytes into the striatum (Figure 1H). On day 3 post-ICH, erythrophagocytic microglia/macrophages were identified by flow cytometry in striatal single-cell suspensions as LIVE/DEAD^-^CD45^int/hi^CD11b^+^-DiD^+^ cells (Figure 1I). Quantitative analysis revealed significantly increased erythrophagocytosis by microglia/macrophages (LIVE/DEAD^-^CD45^int/hi^CD11b^+^-DiD^+^) in ABX mice compared with SPF controls; this effect was reversed following FMT (Figure 1J). Next, to assess hematoma lysis and brain swelling, T2- and T2*-weighted MRI scans were performed on day 3 post-ICH (Figure 1K). Previous studies have confirmed that T2* hyperintense or isointense signals are indicative of erythrolysis 31. Quantification of T2* signal volumes revealed significantly reduced erythrolysis in ABX-treated mice compared with SPF controls. Bilateral ventricular volume was measured using T2-weighted imaging, and the degree of ipsilateral ventricular compression—an indicator of brain edema—was significantly diminished in ABX-treated mice.

Restoration of gut microbiota via FMT reversed the observed reductions in both hematoma lysis and brain edema (Figure 1L, M). Collectively, these findings demonstrate that gut microbiota depletion promotes erythrocyte phagocytosis, inhibits hematoma lysis, and facilitates early hematoma resolution and neurological recovery following ICH.

### Gut microbiota depletion promotes hematoma resolution and neurological recovery via Tregs

**Figure 2:**
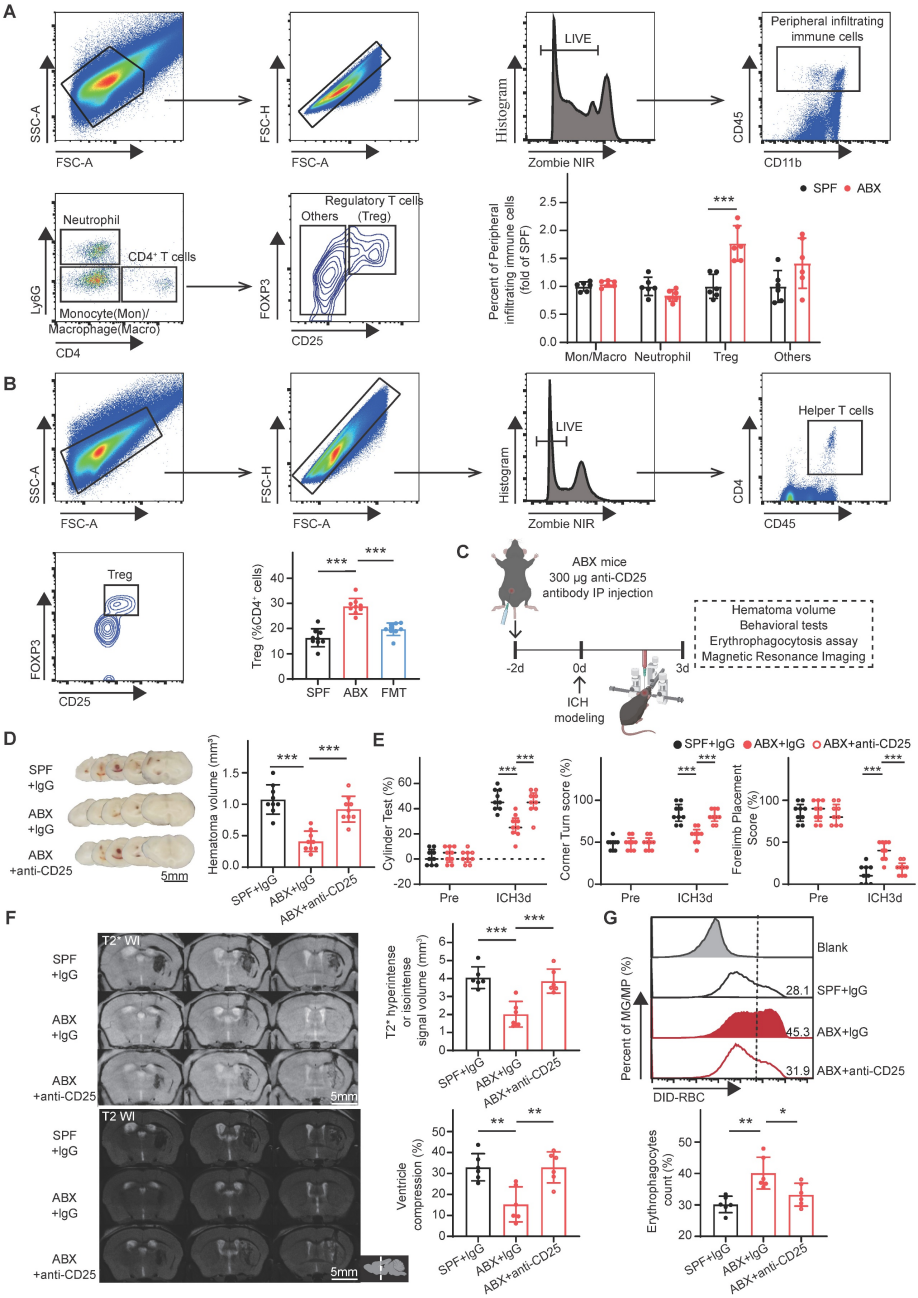
** Gut microbiota depletion increases the brain Treg population to promote hematoma resolution and neurological recovery after ICH.** (A) FACS gating strategy used to isolate neutrophils (LIVE/DEAD^-^CD45^int/hi^CD11b^+^ly6G^+^CD4^-^), monocytes/macrophages (LIVE/DEAD^-^CD45^int/hi^CD11b^+^ly6G^-^CD4^-^), Tregs (LIVE/DEAD^-^CD45^int/h^ly6G^-^CD4^+^CD25^+^Foxp3^+^), and other immune cells (LIVE/DEAD^-^ CD45^int/hi^ly6G^-^CD4^+^CD25^-^Foxp3^-^) among peripheral infiltrating immune cells in the brains of SPF and ABX-treated mice on day 3 post-ICH. Quantification of immune cell populations was performed via flow cytometry. Data are presented as mean ± SD (n = 6 per group). (B) FACS gating strategy used to quantify Tregs (LIVE/DEAD^-^CD45^int/hi^CD4^+^CD25^+^Foxp3^+^) among CD4^+^ T helper cells (LIVE/DEAD^-^CD45^int/hi^CD4^+^) in the brains of SPF, ABX, and FMT mice on day 3 post-ICH. Data are presented as mean ± SD (n = 9 per group). (C) Schematic diagram illustrating the experimental workflow for evaluating the effects of Treg depletion on hematoma resolution, neurological recovery, microglial/macrophage erythrophagocytosis, and hematoma lysis after ICH in ABX-treated mice. Mice received an intraperitoneal injection of anti-CD25 antibody or IgG control 2 days before ICH induction. (D) Representative coronal brain sections from SPF + IgG, ABX + IgG, and ABX + anti-CD25 antibody-treated mice on day 3 post-ICH, showing residual hematomas. Scale bar = 5 mm. Quantification of residual hematoma volume on day 3 post-ICH. Data are presented as mean ± SD (n = 9 per group). (E) Neurological function assessed on day 3 post-ICH in SPF + IgG, ABX + IgG, and ABX + anti-CD25 antibody-treated mice using the cylinder, corner, and forelimb placement tests. Data are presented as median ± IQR (n = 9 per group). (F) Representative T2- and T2*-weighted MRI scans of SPF + IgG, ABX + IgG, and ABX + anti-CD25 antibody-treated groups on day 3 post-ICH. Dashed lines indicate the location of hematoma in the brain. Quantification of T2* hyperintense or isointense lesion volumes and ipsilateral ventricular compression as an indicator of brain swelling. Data are presented as mean ± SD (n = 6 per group). (G) Flow cytometry analysis of erythrophagocytic microglia/macrophages in SPF + IgG, ABX + IgG, and ABX + anti-CD25 antibody-treated groups on day 3 post-ICH. Data are presented as mean ± SD (n = 6 per group). Statistical significance: *p < 0.05, **p < 0.01, ***p < 0.001.

Previous studies have demonstrated that gut microbiota influence neurological outcomes by modulating peripheral immune cells in central nervous system diseases 32-34. Peripheral infiltrating cells, including monocytes/macrophages 9, neutrophils 35, and Tregs 36, play key roles in hematoma resolution after ICH. Therefore, the effects of ABX treatment on brain-infiltrating immune cell populations, including monocytes/macrophages, neutrophils, Tregs, and other subsets, were evaluated following ICH. ABX treatment selectively and substantially increased the number of Tregs in the brain after ICH (Figure 2A). To further explore the regulatory role of gut microbiota in modulating the Treg population post-ICH, FMT was performed. The number of brain-infiltrating Tregs was significantly higher in ABX-treated mice compared with SPF controls, and this increase was abolished following FMT (Figure 2B).

To investigate whether gut microbiota depletion increases the brain Treg population and subsequently accelerates hematoma resolution and neurological recovery after ICH, anti-CD25 monoclonal antibody was administered to deplete Tregs in ABX-treated mice. Experimental groups included SPF mice treated with IgG isotype control and ABX-treated mice administered either IgG or anti-CD25 antibody (Figure 2C). ABX + anti-CD25-treated mice exhibited significantly larger residual hematoma volumes compared with ABX + IgG-treated mice on day 3 post-ICH, with hematoma volumes approaching levels observed in SPF + IgG-treated controls (Figure 2D). Neurological recovery was delayed in ABX + anti-CD25-treated mice relative to ABX + IgG-treated mice and remained comparable to SPF + IgG-treated mice (Figure 2E). Quantitative analysis of MRI signal intensity revealed significantly reduced T2* hyperintense or isointense signal volumes and ipsilateral ventricular compression in ABX + IgG-treated mice compared with SPF + IgG controls. These reductions in erythrocyte lysis and brain swelling were revered by Treg depletion, indicating the loss of the ABX-mediated protective effects (Figure 2F). Flow cytometric analysis of erythrophagocytosis revealed that the increase in microglia/macrophage-mediated phagocytosis observed in ABX + IgG-treated mice was significantly reduced following Treg depletion (Figure 2G). Collectively, these findings indicate that gut microbiota depletion promotes hematoma resolution and neurological recovery after ICH primarily by expanding the Treg population in the brain.

### Tregs promote hematoma resolution and neurological recovery by enhancing scavenger pathway-mediated erythrophagocytosis and suppressing complement-mediated erythrocyte lysis

**Figure 3:**
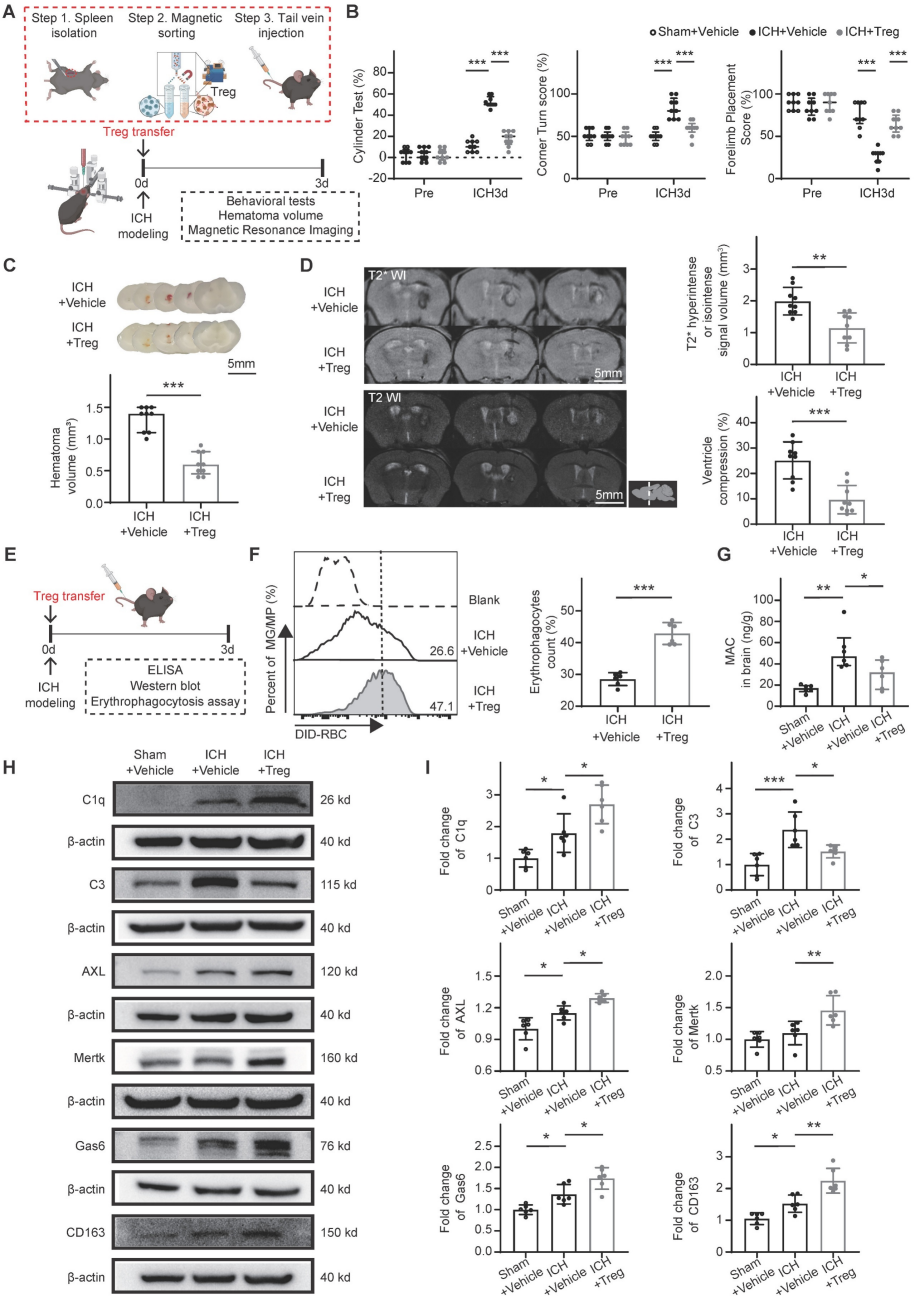
** Tregs promote scavenger pathway-mediated erythrophagocytosis and suppress complement-mediated erythrolysis to facilitate hematoma resolution and neurological recovery.** (A) Schematic diagram illustrating the experimental workflow to assess the effects of adoptive Treg transfer on hematoma resolution, neurological recovery, and hematoma lysis after ICH. Tregs were magnetically isolated from splenocytes of donor mice and administered via tail vein injection to recipient mice immediately after ICH induction. (B) Neurological function assessed in ICH + Vehicle and ICH + Treg mice using the cylinder, corner, and forelimb placement tests on day 3 post-ICH. Data are presented as median ± IQR (n = 9 per group). (C) Representative coronal brain sections showing residual hematomas in ICH + Vehicle and ICH + Treg groups on day 3 post-ICH. Scale bar = 5 mm. Quantification of residual hematoma volume in each group on day 3 post-ICH. Data are presented as median ± IQR (n = 9 per group). (D) Representative T2- and T2*-weighted MRI scans from ICH + Vehicle and ICH + Treg groups on day 3 post-ICH. T2* hyperintense or isointense lesion volumes were quantified, and ipsilateral ventricular compression was measured as an indicator of brain swelling. Data are presented as mean ± SD (n = 9 per group). (E) Schematic diagram illustrating the workflow to evaluate the effects of adoptive Treg transfer on microglial/macrophage erythrophagocytosis and associated downstream molecular markers. Tregs were transferred into recipient mice via tail vein injection after ICH. (F) Percentages numbers of erythrophagocytic microglia/macrophages assessed by flow cytometry in ICH + Vehicle and ICH + Treg groups on day 3 post-ICH. Data are presented as mean ± SD (n = 6 per group). (G) Membrane attack complex (MAC) content measured in perihematomal brain tissue of Sham + Vehicle, ICH + Vehicle, and ICH + Treg groups on day 3 post-ICH using ELISA. Data are presented as median ± IQR (n = 6 per group). (H) Western blot analysis of C1q, C3, AXL, MERTK, Gas6, CD163, and β-actin protein levels in perihematomal brain tissue from Sham + Vehicle, ICH + Vehicle, and ICH + Treg groups on day 3 post-ICH. (I) Densitometric quantification of C1q, C3, AXL, Mertk, Gas6, and CD163 protein levels based on Western blot results. Data are presented as mean ± SD (n = 6 per group). Statistical significance: *p < 0.05, **p < 0.01, ***p < 0.001.

To further evaluate the role of Tregs in hematoma resolution following ICH, Tregs were isolated from donor mice and transferred into recipient mice after ICH induction to increase the brain Treg population. Functional outcomes, erythrophagocytosis, hematoma lysis, and associated downstream molecular markers were assessed in the ICH + Vehicle and ICH + Treg groups (Figure 3A). The ICH + Treg group exhibited improved neurological performance and markedly reduced residual hematoma volume compared with the ICH + Vehicle group (Figure 3B, C). MRI analysis demonstrated that both erythrocyte lysis and brain edema were significantly attenuated in the ICH + Treg group (Figure 3D).

Following adoptive Treg transfer, erythrophagocytosis and associated downstream molecular markers were assessed in both groups (Figure 3E). Flow cytometry analysis revealed a significantly higher proportion of erythrophagocytic microglia/macrophages in the ICH + Treg group than in the ICH + Vehicle group (Figure 3F). Previous studies have demonstrated that complement component C3 activation and MAC formation contribute to erythrolysis 7, 31, while C1q/MERTK/AXL-Gas6 and hemoglobin-CD163 represent the primary scavenger pathways involved in erythrophagocytosis 9. Accordingly, key downstream molecular markers related to erythrophagocytosis and erythrocyte lysis were evaluated. Treg treatment significantly reduced MAC formation and C3 protein expression, while substantially increasing the expression levels of C1q, MERTK, AXL, Gas6, and CD163 compared with vehicle treatment (Figure 3G-I). These findings indicate that Tregs promote hematoma resolution by enhancing scavenger pathway-mediated erythrocyte clearance and suppressing complement-mediated erythrolysis.

### Gut microbiota depletion triggers Treg production in the gut and spleen

**Figure 4:**
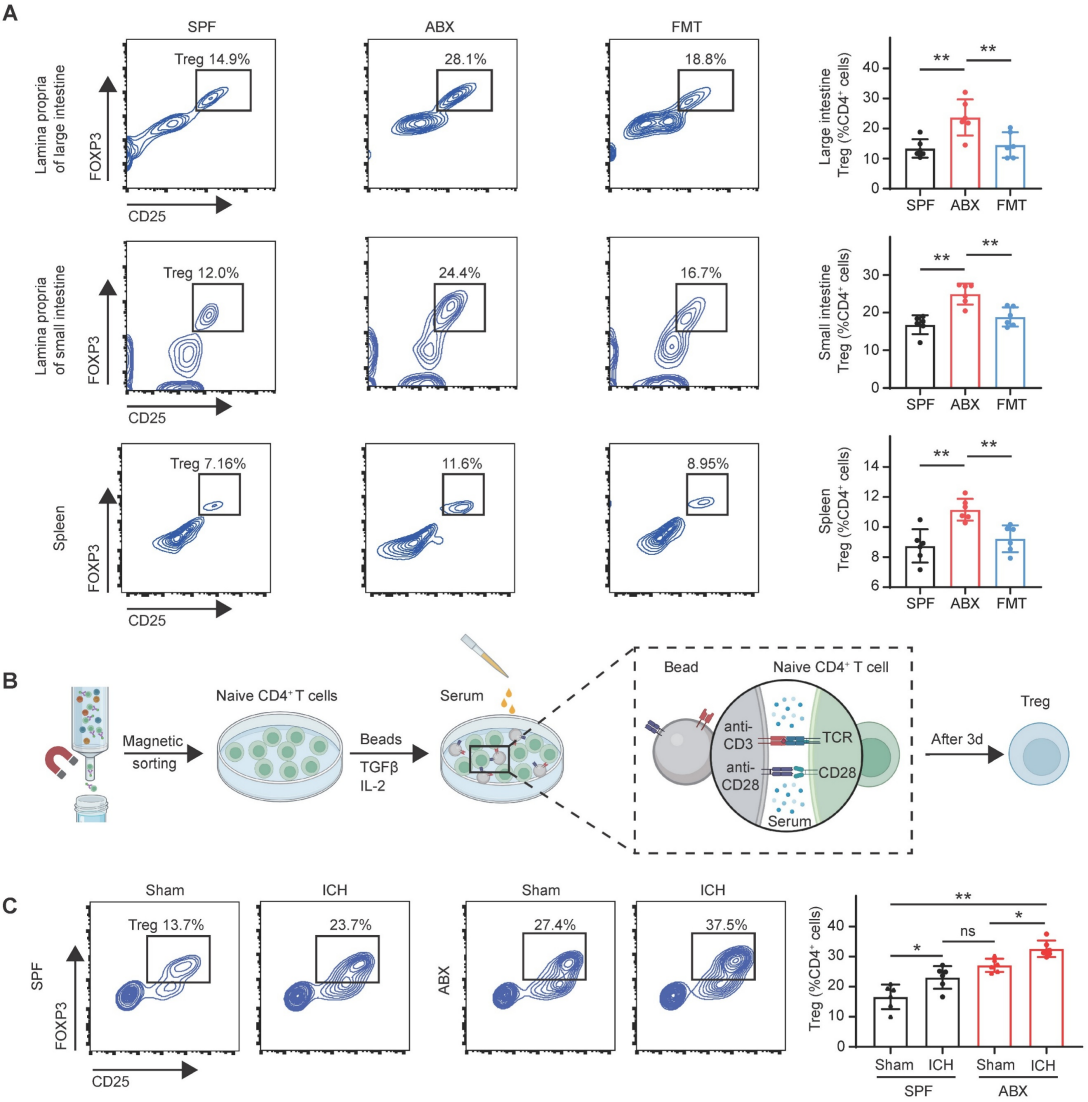
** Gut microbiota depletion triggers Treg production in the gut and spleen.** (A) Percentages of Tregs in the lamina propria of large intestine, small intestine, and spleen in SPF, ABX, and FMT groups, determined by flow cytometry on day 3 post-ICH. Representative contour plots represent Treg populations in the lamina propria of the large intestine (top), small intestine (middle), and spleen (bottom). Data are presented as mean ± SD (n = 6 per group). (B) Schematic diagram illustrating the experimental workflow for assessing the effects of serum-derived factors from different experimental groups on Naïve CD4^+^ T cells differentiation into Tregs. Naïve CD4^+^ T cells were magnetically sorted from splenocytes and cultured under suboptimal Treg-inducing conditions (0.1 ng/mL TGF-β1, 10 ng/mL IL-2, CD3-T cell receptor [TCR] and anti-CD28 signaling) with serum from the following groups: SPF + Sham, SPF + ICH, ABX + Sham, and ABX + ICH. Treg differentiation was analyzed by flow cytometry on day 3. (C) Percentages of Tregs generated *in vitro* under the influence of serum from SPF + Sham, SPF + ICH, ABX + Sham, and ABX + ICH groups as determined by flow cytometry. Representative contour plots represent Treg populations in each group. Data are presented as mean ± SD (n = 6 per group).

Tregs—primarily distributed in the spleen, thymus, and gut—have been shown to infiltrate and proliferate in the brain during central nervous system diseases 37-39. To determine whether the increased brain Treg population observed in ABX-treated mice after ICH resulted from enhanced proliferation, Ki67^+^ Tregs were quantified in the brain. Flow cytometry analysis revealed no significant differences in Ki67^+^ Treg populations among the SPF, ABX, and FMT groups (Figure S3A), indicating that gut microbiota depletion did not significantly alter Treg proliferative capacity in the brain. A previous study by our group reported T cell migration from the intestine to the perihematomal region following ICH 23, consistent with other reports demonstrating that immune cells originating from the intestine and spleen can migrate into the brain following stroke 40, 41. To investigate whether gut microbiota regulate Treg populations in the spleen, thymus, and intestine, thereby contributing to Treg expansion in the brain, Treg numbers were quantified in the thymus, spleen, intestine, and blood of SPF, ABX, and FMT mice after ICH. Flow cytometry analysis revealed significant increases in Treg populations in the lamina propria of both the small and large intestines, as well as in the spleen, of ABX-treated mice compared with SPF controls. However, FMT reversed these increases in ABX-treated mice following ICH (Figure 4A). In contrast, Treg populations in the thymus and blood did not differ significantly among the SPF, ABX, and FMT groups (Figure S3B). These results suggest that gut microbiota depletion increases Treg numbers specifically in the intestine and spleen, contributing to Treg accumulation in the brain after ICH.

To elucidate the mechanism by which gut microbiota depletion increases Treg numbers in these peripheral organs, Treg proliferation was assessed by quantifying Ki67^+^ Tregs in the intestine and spleen. Although Tregs arise through differentiation of naïve CD4^+^ T cells under the influence of specific stimuli, such as the cytokine TGF-β and microbial metabolites derived from bile acids, and subsequently expand via proliferation 42, no significant differences in Ki67^+^ Treg populations were observed in the lamina propria of the small and large intestines or in the spleen among SPF, ABX, and FMT groups after ICH (Figure S3C). These findings suggest that the expansion of Tregs following gut microbiota depletion is not driven by increased proliferation. Prior studies have shown that microbial metabolites play key roles in regulating Treg differentiation 17, 21, 43, supporting the hypothesis that gut microbiota depletion promotes Treg generation through increased differentiation of naïve CD4^+^ T cells. To validate this hypothesis, naïve CD4^+^ T cells were magnetically sorted from the spleens of SPF mice and treated with sera collected from four experimental groups: SPF mice subjected to sham surgery (SPF + Sham), SPF mice subjected to ICH (SPF + ICH), ABX-treated mice subjected to sham surgery (ABX + Sham), and ABX-treated mice subjected to ICH (ABX + ICH) (Figure 4B). Serum derived from ICH mice promoted greater differentiation of naïve CD4^+^ T cells into Tregs than serum from sham-operated mice in both SPF and ABX groups.

Furthermore, serum from ABX-treated mice induced significantly higher levels of Treg differentiation compared with serum from SPF mice under both Sham and ICH conditions (Figure 4C). These findings indicate that serum-derived factors from ABX-treated mice promote differentiation of naïve CD4^+^ T cells into Tregs, leading to increased Treg populations in the intestine and spleen after ICH.

### Gut microbiota depletion increases serum and fecal PCA to enhance Treg differentiation via the PKCθ-AKT-FoxO1/3a pathway

**Figure 5:**
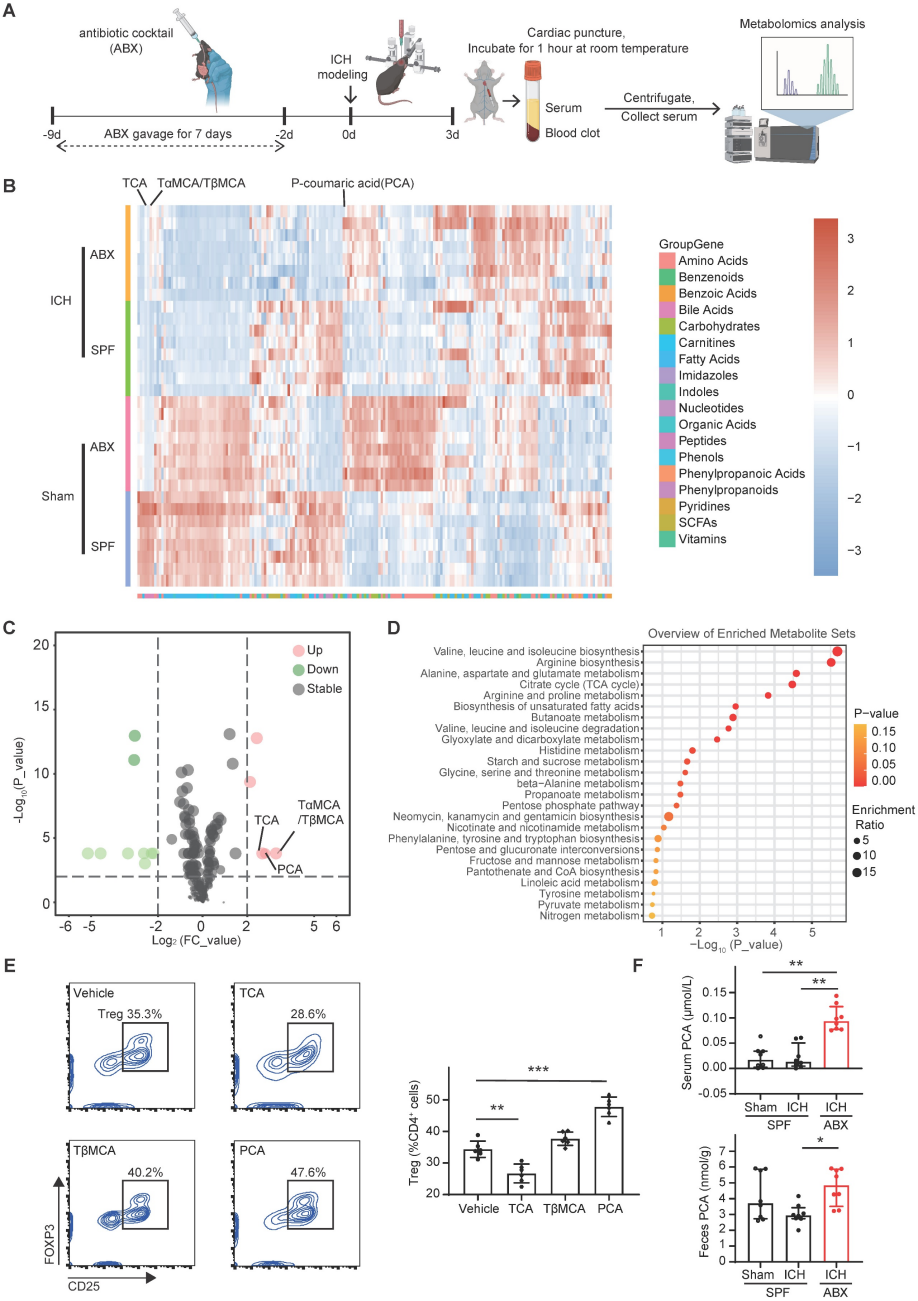
** Gut microbiota depletion enhances PCA levels in serum and feces, promoting Treg differentiation.** (A) Schematic diagram illustrating the experimental workflow for serum metabolomic profiling. Blood samples were collected via cardiac puncture from ABX-treated mice on day 3 post-ICH and incubated at room temperature for 1 hour. Serum was isolated by centrifugation and subjected to targeted metabolomic analysis. (B, C) Heatmaps and volcano plots showing differential serum metabolite profiles among SPF + Sham, SPF + ICH, ABX + Sham, and ABX + ICH groups on day 3 post-ICH. The top three upregulated metabolites in ABX-treated mice compared with SPF mice were TCA, Tα/βMCA, and PCA (n = 8 per group). (D) Kyoto Encyclopedia of Genes and Genomes (KEGG) pathway enrichment analysis of differentially expressed serum metabolites between SPF and ABX groups on day 3 post-ICH (n = 8 per group). Statistical analysis: ANOVA followed by Bonferroni post hoc tests in (B); and Student's t-test in (C-D). Fold-change scale adjusted to 4; Uni *p* < 0.05 considered statistically significant (B-D). (E) Naïve CD4^+^ T cells were cultured with Vehicle, TCA, TβMCA, or PCA under Treg-inducing conditions. Percentages of Tregs generated in each treatment group were determined by flow cytometry. Representative contour plots represent percentages of generated Tregs in each group. Data are presented as mean ± SD (n = 6 per group). (F) PCA concentrations in feces and serum of SPF + Sham, SPF + ICH, and ABX + ICH groups on day 3 post-ICH. Data are presented as median ± IQR (n = 8 per group).

To identify serum-derived factors responsible for triggering naïve CD4^+^ T cells differentiation into Tregs in ABX-treated mice, metabolomic profiling was performed on serum samples collected from four experimental groups: SPF + Sham, ABX + Sham, SPF + ICH, and ABX + ICH (Figure 5A). Principal component analysis revealed distinct clustering between groups, indicating significant metabolomic differences (Figure S4). Heatmap and volcano plot analysis identified TαMCA/TβMCA, PCA, and TCA as the top three upregulated metabolites in the serum of ABX-treated mice compared with SPF controls after ICH (Figure 5B, C). Kyoto Encyclopedia of Genes and Genomes (KEGG) pathway enrichment analysis furtherly identified valine, leucine, and isoleucine biosynthesis; arginine biosynthesis; and alanine, aspartate, and glutamate metabolism as the top three upregulated metabolic pathways in the serum of ABX + ICH group compared with the SPF + ICH group (Figure 5D). To assess whether these upregulated metabolites influence Treg differentiation, naïve CD4^+^ T cells were treated *in vitro* with vehicle, TCA, TβMCA, or PCA. Among the tested metabolites, PCA significantly enhanced the differentiation of naïve CD4^+^ T cells into Tregs, while TCA substantially inhibited Treg differentiation. TβMCA did not significantly alter Treg differentiation compared with vehicle (Figure 5E). Mass spectrometry analysis confirmed that both serum and fecal PCA concentrations were significantly elevated in ABX-treated mice compared with SPF controls after ICH (Figure 5F). These findings suggest that gut microbiota depletion promotes Treg differentiation by increasing PCA levels in both serum and feces.

**Figure 6:**
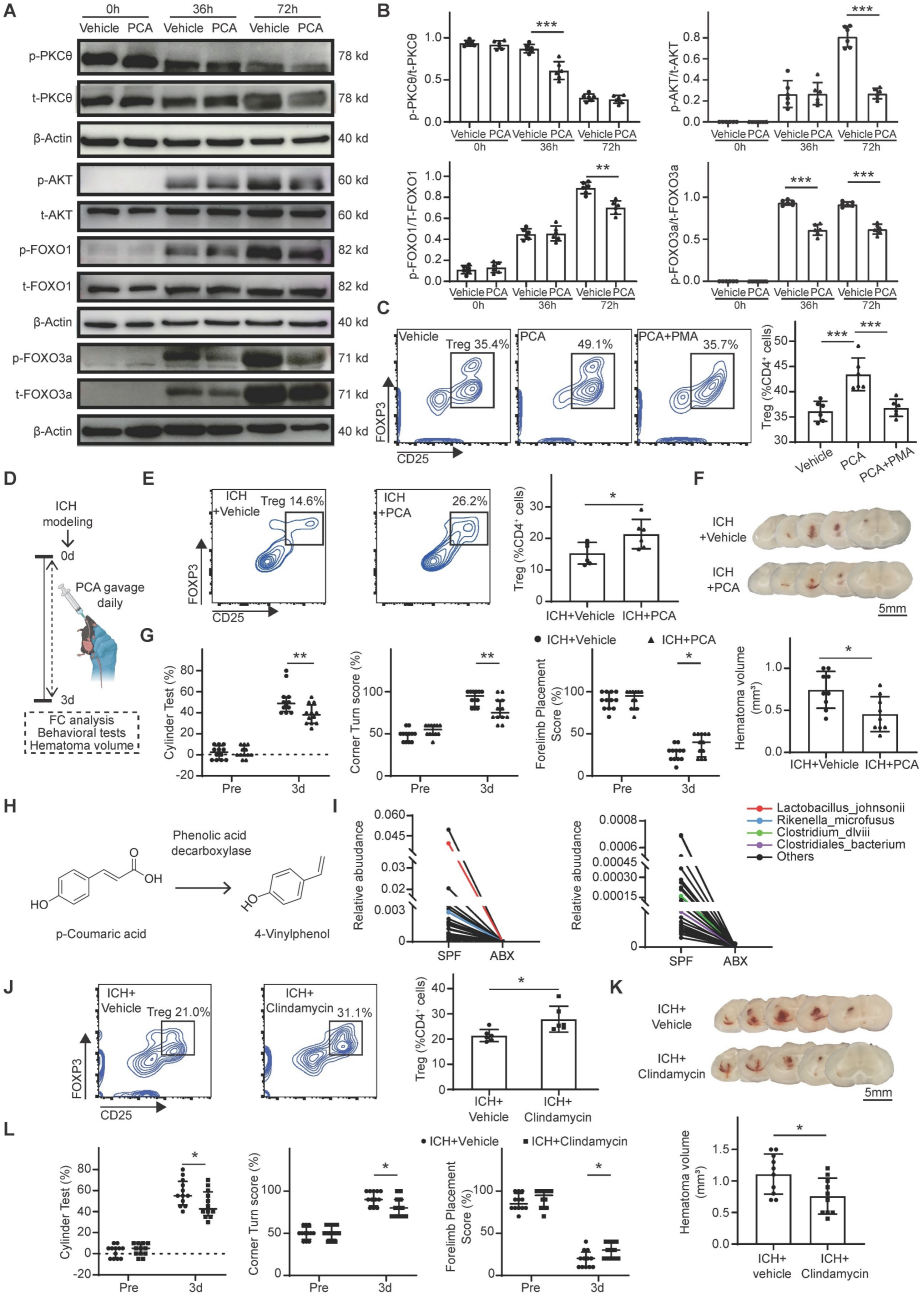
** p-Coumaric acid (PCA) triggers Treg differentiation via the PKCθ-AKT-FoxO1/3a pathway.** Oral gavage of PCA or clindamycin to deplete PCA-metabolizing microbiota increases the brain Treg population, facilitates hematoma resolution, and accelerates neurological recovery after ICH. (A) Western blot analysis of naïve CD4^+^ T cells treated with Vehicle or PCA for 0, 36, and 72 hours. Target proteins included p-PKCθ, total PKCθ, p-AKT, total AKT, p-FoxO1, total FoxO1, p-FoxO3a, total FoxO3a, and β-actin. (B) Quantitative analysis of p-PKCθ to total PKCθ, p-AKT to total AKT, p-FoxO1 to total FoxO1, and p-FoxO3a to total FoxO3a ratios based on Western blot analysis. Data are presented as mean ± SD (n = 6 per group). (C) Flow cytometry analysis of Treg differentiation in naïve CD4^+^ T cells treated with Vehicle, PCA, or PCA + PMA. Percentages of generated Tregs in each treatment group are shown with representative contour plots. Data are presented as mean ± SD (n = 6 per group). (D) Schematic diagram illustrating the experimental workflow to assess the effects of PCA oral gavage on brain Treg population, hematoma resolution and neurological recovery after ICH. (E) Percentages of Tregs in the brains of ICH + Vehicle and ICH + PCA groups, determined by flow cytometry. Representative contour plots represent percentages of Tregs in the brains of ICH + Vehicle and ICH + PCA groups. Data are presented as mean ± SD (n = 6 per group). (F) Representative coronal brain sections showing residual hematomas in ICH + Vehicle and ICH + PCA groups on day 3 post-ICH. Scale bar = 5 mm. Quantification of residual hematoma volume in ICH + Vehicle and ICH + PCA groups on day 3 post-ICH. Data are presented as mean ± SD (n = 9 per group). (G) Neurological function assessed using the cylinder, corner, and forelimb placement tests in ICH + Vehicle and ICH + PCA groups. Data are presented as median ± IQR (n = 12 per group). (H) Proposed metabolic pathway of PCA metabolism via phenolic acid decarboxylase, an enzyme expressed by specific gut bacterial species. (I) Line graph illustrating reduced relative abundance of four PCA-metabolizing bacterial species in ABX-treated mice compared with SPF mice on day 3 post-ICH (p < 0.05). Red, blue, purple, and green lines represent *Lactobacillus johnsonii*, *Rikenella microfusus*, *Clostridiales bacterium*, and *Clostridium dlviii*, respectively. (J) Percentages of Tregs in the brains of ICH + Vehicle and ICH + Clindamycin groups on day 3 post-ICH, determined by flow cytometry. Representative contour plots represent percentages of Tregs in the brains of ICH + Vehicle and ICH + Clindamycin mice. Data are presented as mean ± SD (n = 6 per group). (K) Representative coronal brain sections from ICH + Vehicle and ICH + Clindamycin groups on day 3 post-ICH showing residual hematomas. Scale bar = 5 mm. Quantification of residual hematoma volume on day 3 post-ICH. Data are presented as mean ± SD (n = 10 per group). (L) Neurological function assessed by the cylinder, corner, and forelimb placement tests in ICH + Vehicle and ICH + Clindamycin groups. Data are presented as median ± IQR (n = 12 per group). Statistical significance: *p < 0.05, **p < 0.01, ***p < 0.001.

The downstream signaling mechanism by which PCA induces Treg differentiation was next investigated. PCA has been reported to inhibit T cell activation through suppression of PKCθ phosphorylation 44. PKCθ impedes Treg differentiation by activating the PKCθ-AKT-FoxO1/3a pathway 45. To determine whether PCA promotes Treg differentiation via inhibition of this pathway, naïve CD4^+^ T cells were treated with vehicle or PCA under Treg-priming conditions for 0, 36, and 72 hours, followed by Western blot analysis of phosphorylated and total PKCθ, AKT, and FoxO1/3a. In the Vehicle group of naïve CD4^+^ T cells, phosphorylated PKCθ expression levels gradually declined from 0 to 72 hours, while phosphorylated AKT and FoxO1/3a levels progressively increased. In contrast, PCA-treated cells exhibited a lower ratio of phosphorylated to total PKCθ at 36 hours, and reduced phosphorylation of AKT and FoxO1 at 72 hours. PCA treatment also significantly suppressed FoxO3a phosphorylation in naïve CD4^+^ T cells at both 36 and 72 hours compared with vehicle (Figure 6A, B). To directly assess whether PCA promotes Treg differentiation via inhibition of the PKCθ-AKT-FoxO1/3a pathway, naïve CD4^+^ T cells were co-treated with PCA and PMA, a known PKCθ activator 45, 46, for 3 days. Flow cytometry analysis revealed that co-treatment with PMA abolished the Treg-promoting effect of PCA, indicating that PKCθ activation negated the influence of PCA on Treg differentiation (Figure 6C). These findings suggest that PCA facilitates the differentiation of naïve CD4^+^ T cells into Tregs by suppressing the PKCθ-AKT-FoxO1/3a signaling axis, thereby linking gut microbiota-derived metabolites to immune modulation in the context of ICH.

### PCA and PCA-metabolizing microbiota depletion enhance brain Treg population, promote hematoma resolution, and accelerate neurological recovery after ICH

To confirm that PCA increases the Treg population in the brain and thereby promotes hematoma resolution and neurological recovery after ICH *in vivo*, PCA or vehicle was administered via oral gavage daily for 3 consecutive days following ICH induction. Neurological deficits, hematoma volume, and brain Treg populations were assessed in the vehicle and PCA-treated groups (Figure 6D). Flow cytometry analysis revealed a significantly increased Treg population in the brain of PCA-treated mice compared with vehicle-treated controls on day 3 post-ICH (Figure 6E). PCA treatment also resulted in a smaller residual hematoma volume and improved neurological function relative to vehicle treatment (Figure 6F, G). PCA is metabolized by certain gut bacteria into 4-vinylphenol through the activity of phenolic acid decarboxylase (Figure 6H) 47. Based on this, it was hypothesized that increased PCA levels in serum and feces following ABX treatment were attributable to depletion of PCA-metabolizing microbiota. To test this hypothesis, 16S rRNA gene sequencing was performed on fecal samples from SPF and ABX mice after ICH. Analysis revealed markedly lower abundances of *Lactobacillus johnsonii*, *Rikenella microfusus*, *Clostridiales bacterium*, and *Clostridium dlviii*—bacteria species known to express phenolic acid decarboxylase 48, 49—in ABX-treated mice compared with SPF controls (Figure 6I).

Next, the effects of PCA-metabolizing microbiota depletion on the brain Treg population, hematoma resolution, and neurological recovery was examined. *L. johnsonii*, *R. microfusus*, *C. bacterium*, and *C. dlviii* are anaerobic bacteria. Metronidazole is the first-line agent for anaerobic bacterial infections but is contraindicated in ICH due to its neurotoxicity and potential to induce encephalopathy 50. Clindamycin, a lincosamide antibiotic with broad-spectrum efficacy against anaerobes, was selected as an alternative. Clindamycin or vehicle was administered to ICH mice for 3 days to deplete PCA-metabolizing microbiota. Behavioral assessments, hematoma volume quantifications, and flow cytometry were performed on day 3 post-ICH in the vehicle and clindamycin-treated groups. Flow cytometry analysis revealed a significantly higher Treg population in the brain of clindamycin-treated mice compared with the Vehicle group (Figure 6J). Clindamycin administration also led to a reduction in hematoma volume and significantly improved neurological function relative to vehicle controls on day 3 post-ICH (Figure 6K, L).

## Discussion

This study uncovered previously unreported roles for gut microbiota in regulating hematoma resolution and Treg production following ICH. The key findings were as follows: (1) Gut microbiota depletion significantly accelerated hematoma resolution and neurological recovery after ICH by enhancing microglia/macrophage erythrophagocytosis and suppressing hematoma lysis. (2) Elimination of gut microbiota increased the brain Treg population post-ICH, contributing to accelerated hematoma resolution. (3) Adoptive transfer of Tregs promoted scavenger pathway-mediated erythrophagocytosis and inhibited complement-mediated hematoma lysis by upregulating C1q, MERTK, AXL, Gas6, and CD163 expression, while downregulating C3 expression and MAC formation. (4) Gut microbiota depletion elevated PCA concentrations in serum and feces, facilitating differentiation of naïve CD4^+^ T cells into Tregs via suppression of the PKCθ-AKT-FoxO1/3a signaling pathway. (5) Both PCA administration and clindamycin-induced depletion of PCA-metabolizing microbiota augmented the brain Treg population, enhanced hematoma resolution, and improved neurological recovery. These results highlight the therapeutic potential of gut microbiota modulation and Treg-based strategies in the treatment of ICH.

Post-ICH mechanisms of brain injury highlight the importance of mitigating hematoma-induced damage to the brain parenchyma, as all downstream pathological processes originate from the hematoma itself. Cerebral hematoma resolution remains the most promising therapeutic strategy for ICH management and therefore warrants increased focus. Post-ICH hematoma resolution is governed by two key factors: the rate of hematoma lysis and the efficiency of hematoma clearance by microglia/macrophages 30. While extensive research has addressed phagocytic activity of microglia/macrophages and scavenger pathway-mediated erythrophagocytosis, the regulation of hematoma lysis has received comparatively limited attention 8, 51-54. Complement-mediated hematoma lysis leads to the release of hemoglobin and heme, which contribute to perihematomal neuronal injury prior to hematoma clearance post-ICH 7, 55. Therefore, the dual approach of enhancing scavenger pathway-mediated clearance while suppressing complement-driven hematoma lysis presents a compelling therapeutic avenue to accelerate hematoma resolution post-ICH. Previous studies have demonstrated that Tregs are involved in regulating both phagocytosis and complement activity. Tregs promote macrophage efferocytosis through IL-13 secretion, which stimulates IL-10 production in macrophages. This IL-10, via autocrine-paracrine signaling, induces vav guanine nucleotide exchange factor 1 (Vav1) expression in macrophages, leading to activation of Ras-related C3 botulinum toxin substrate 1 (Rac1) and enhanced engulfment of apoptotic cells 56. In addition to this established mechanism, the present study demonstrated that Tregs enhance erythrocyte efferocytosis by upregulating the expression of efferocytosis receptors MERTK/AXL and ligands Gas6 and C1q. CD163, a hemoglobin scavenger receptor and M2 microglial polarization marker, plays a crucial role in hematoma resolution 57. A mouse model of ICH previously reported that Tregs promote M2 microglia/macrophage polarization through increased expression of the M2 marker CD206 15. The current findings provide direct evidence that Tregs enhance post-ICH hematoma clearance by increasing CD163 expression levels in the brain.

In a murine model of Alzheimer's-like amyloid pathology, Treg depletion was shown to alter reactive astrocyte subtype distribution, leading to an increase in C3-positive A1-like phenotypes. Consistent with this observation, the present study demonstrated that Tregs suppress complement C3 expression and MAC formation, thereby reducing complement-mediated hematoma lysis after ICH. These findings suggest that therapeutic strategies aimed at enhancing the brain Treg population may facilitate hematoma resolution and represent a promising avenue for ICH treatment.

Gut microbiota play a critical role in the development of Tregs. CD4^+^CD25^+^ T cells isolated from germ-free mice exhibit lower Foxp3 gene expression and reduced suppressive capacity *in vitro* compared with those from SPF mice 58. Exposure to commensal microbiota induces the peripheral generation of Tregs 59. Moreover, gut microbiota produce extracellular vesicles, bacterial DNA, and metabolites—including short-chain fatty acids, indole, and bile acids—all of which contribute to Treg expansion 60. Previous studies have consistently reported that the presence of gut microbiota supports Treg differentiation. In contrast, our present study showed that depletion of gut microbiota via ABX gavage significantly increased the Treg population in a mouse model of ICH. To address the apparent discrepancy between these findings and prior literature, serum metabolite profiling was conducted to identify effector molecules responsible for Treg expansion following gut microbiota depletion. Among the identified metabolites, PCA emerged as a key factor promoting Treg differentiation, as confirmed by *in vitro* differentiation assays. PCA is a phenolic compound commonly found in fruits, vegetables, and grains 61. After ingestion, PCA is partially absorbed into the circulation, while the remainder undergoes microbial metabolism by gut bacteria expressing phenolic acid decarboxylase 47. Fecal 16S rRNA sequencing and mass spectrometry analysis of fecal and serum samples from ABX-treated and SPF mice after ICH revealed that ABX gavage elevated PCA levels in feces and serum by eliminating PCA-metabolizing bacteria, thereby facilitating Treg differentiation. These results support the conclusion that, beyond the production of Treg-promoting metabolites, gut microbiota regulate Treg differentiation by modulating the availability of these metabolites through microbial consumption. This discovery introduces a novel approach to enhance Treg induction through targeted antibiotic-mediated depletion of specific microbial populations. In the present study, treatment with clindamycin effectively depleted PCA-metabolizing bacteria and resulted in a significant increase in the brain Treg population relative to vehicle-treated mice. Additionally, ABX oral gavage for 1 week was introduced as an efficient method for gut microbiota depletion in the context of ICH. Compared with the conventional 4-week ABX administration via drinking water, the 1-week oral gavage approach achieved rapid and robust microbial depletion. Notably, conclusions derived from ABX-treated mice are more translationally relevant than those from germ-free models, as human subjects are not maintained in germ-free environments.

Foxp3^+^ Tregs mainly originate in the thymus (thymic Tregs, tTregs) but can also be generated extrathymically at peripheral sites (pTregs) 62. tTregs develop within the thymus through T cell receptor interactions with self-peptide/major histocompatibility complexes exhibiting relatively high affinity 63. In contrast, pTregs arise in peripheral tissues, such as the intestine and spleen, from conventional CD4^+^ T cells following T cell receptor stimulation in the presence of TGF-β and IL-2 64. Gut microbiota support pTreg production through the action of microbiota-derived metabolites, including bile acids and butyrate 17. However, the relationship between gut microbiota and tTreg development remains unclear. GPR109A, a G-protein-coupled receptor, is activated by bacterially derived short-chain fatty acids such as butyrate and niacin, thereby inducing gut-derived Treg differentiation 65, 66. Notably, the absence of GPR109A increases tTreg generation 67, although no direct evidence has established a mechanistic link between gut microbiota and tTregs. The present study demonstrated that gut microbiota depletion triggers pTreg differentiation in the gut and spleen by increasing PCA levels, consistent with previously reported pathways of metabolite-mediated Treg induction. The absence of substantial differences in tTreg populations between ABX-treated and SPF mice after ICH suggests that gut microbiota depletion post-ICH has minimal impact on tTreg dynamics. These results indicate that targeted regulation of pTregs, rather than tTregs, represents a feasible strategy for expanding the Treg population to facilitate recovery following ICH.

A clinical study demonstrated that ICH induces gut dysbiosis, with alterations in gut microbiota diversity among patients being associated with prognosis 68. A preclinical investigation showed that recolonization of ICH mice with healthy gut microbiota reduced neuroinflammation 23. Additionally, neuroinflammation was substantially reduced in ICH mice following transplantation of microbiota from berberine-treated donors 69. Despite these findings, the precise mechanisms by which gut microbiota influence ICH outcomes remain undefined. In particular, the role of gut microbiota in hematoma resolution, which is a critical determinant of ICH recovery, has not been previously determined. To the best of current knowledge, this study represents the first investigation of the effects and underlying mechanisms of gut microbiota on hematoma resolution and neurological deficits following ICH. Results from this study demonstrated that gut microbiota depletion via ABX gavage facilitated hematoma resolution and improved neurological recovery after ICH. Mechanistic analysis revealed that ABX treatment eliminated phenolic acid decarboxylase-expressing gut bacteria responsible for metabolizing PCA, leading to increased fecal and serum PCA levels. Elevated PCA concentrations promoted Treg differentiation through suppression of the PKCθ-AKT-FoxO1/3a pathway, thereby accelerating hematoma resolution and neurological recovery after ICH. Together with previous evidence, these findings establish that gut microbiota play a functional role in modulating hematoma resolution and shaping ICH outcomes, and that dysregulation of microbial composition may serve as a therapeutic target. Given that gut microbiota depletion in this study was induced by ABX gavage prior to ICH onset, the potential for post-ICH therapeutic application was also examined. Administration of PCA and clindamycin-mediated depletion of PCA-metabolizing microbiota after ICH both resulted in accelerated hematoma resolution and improved neurological recovery. These results support the concept that antibiotic-based modulation of gut microbiota may serve as a viable post-ICH therapeutic strategy.

Furthermore, gut microbiota depletion was found to accelerate hematoma resolution and improve neurological recovery on day 3 after ICH. However, no significant differences in residual hematoma volume or behavioral scores were observed between the SPF and ABX groups on day 7 after ICH. We investigate the underlying cause of this diminished effect. A previous study reported a rebound in fecal bacterial density 1 week after the cessation of ABX treatment 70. In this study, ABX treatment was administered for 1 week prior to ICH induction. Discontinuation of ABX treatment following ICH likely allowed for partial recolonization of the gut microbiota in the ABX group, which may have contributed to insufficient hematoma resolution by day 7. This partial recolonization is a probable explanation for the absence of significant differences in neurological outcomes between the two groups at this later time point. To exclude the possibility of gut microbiota recolonization in ABX mice, future studies will aim to employ germ-free mice to more definitively evaluate the sustained effects of gut microbiota absence on hematoma resolution and neurological recovery after ICH.

Although the present study provided substantial evidence that gut microbiota depletion promotes hematoma resolution and neurological recovery via PCA-triggered Treg differentiation, several limitations should be acknowledged. First, Tregs were shown to enhance efferocytosis by upregulating the expression of MERTK/AXL receptors, Gas6/C1q ligands, and the hemoglobin scavenger receptor CD163, while downregulating complement C3 expression levels in the brain. These effects collectively promoted erythrocyte phagocytosis and suppressed clot lysis following ICH. However, the precise molecular mechanisms by which Tregs regulate complement activation and efferocytosis remained undefined. Second, clindamycin was used to investigate the role of PCA-metabolizing bacteria after ICH, but the specific functions of these microbial species have not yet been determined. Future investigations should assess hematoma resolution and neurological outcomes in germ-free mice recolonized with these bacteria to clarify their individual contributions. Third, sex-related differences, including the influence of estrogen levels on ICH outcomes and gut microbiota composition, were not addressed in this study, which utilized only male mice. Subsequent research should examine the therapeutic potential of gut microbiota modulation in female ICH models to ensure broader applicability of findings.

## Conclusion

The results of this study provide strong evidence that gut microbiota depletion accelerates hematoma resolution and promotes neurological recovery through PCA-mediated Treg differentiation, highlighting a novel immunometabolic axis with therapeutic relevance in ICH.

## Supplementary Material

Supplementary figures and table.

## Abbreviations

ICH: Intracerebral hemorrhage
Treg: Regulatory T cell
ABX: Antibiotic cocktail
pTreg: Peripheral Treg
PCA: p-Coumaric acid
SPF: Specific pathogen-free
Foxp3: Forkhead box p3
FMT: Fecal microbiota transplantation
PKCθ: Protein kinase C-theta
AKT: Protein kinase B
FoxO1/3a: Forkhead box O1/3a
TαMCA/TβMCA: Tauro-α/β-muricholic acid
TCA: Taurocholic acid
PMA: Phorbol-12-myristate-13-acetate
tTreg: Thymus regulatory t cell
