# Supplementary material for: Gut microbiota depletion accelerates hematoma resolution and neurological recovery after intracerebral hemorrhage via p-coumaric acid-promoted Treg differentiation
Source: Theranostics. 2025 Jun 9;15(14):6628–50. doi: 10.7150/thno.113764 (PMC12203673; doi:10.7150/thno.113764)
Supplement: Supplementary file 1 — Supplementary figures and table. [file thnov15p6628s1.pdf]

Figure S1: FACS gating strategy used to isolate naïve $CD4^+$ T cells ($CD45^+CD4^+CD25^-$ $CD44^{low}CD62L^{high}$).

Figure S2: (A) Linear discriminant analysis effect size (LEfSe) showing statistically significant differences in gut microbiota composition between SPF and ABX groups. Taxa with LDA threshold > 4 are shown (n = 5 per group). (B) Bacterial taxonomic composition at the genus level in SPF and ABX mice on day 3 post-ICH (n = 5 per group).

Figure S3: Treg proliferation and tissue distribution following after ICH. (A) FACS gating strategy to isolate proliferating $Ki67^+$ Tregs ($LIVE/DEAD^-CD45^{int/hi}CD4^+CD25^+Foxp3^+Ki67^+$). Percentages of $Ki67^+$ Tregs in the brain on day 3 post-ICH, assessed in SPF, ABX, and FMT groups. Data are presented as mean ± SD (n = 6 per group). (B) Percentages of Tregs in the thymus (top) and blood (bottom) of SPF, ABX, and FMT groups, determined by flow cytometry. Data are presented as mean ± SD (n = 6 per group). (C) Percentages of $Ki67^+$ Tregs in the lamina propria of the large intestine (left), small intestine (middle), and spleen (right) on day 3 post-ICH, assessed in SPF, ABX, and FMT groups. Data are presented as mean ± SD (n = 6 per group). ns = not significant.

Figure S4: Principal component analysis of serum metabolites in SPF + Sham, SPF + ICH, ABX + Sham, and ABX + ICH mice (n = 8 per group), showing distinct separation among groups. ANOVA followed by Bonferroni post hoc test was used to determine significance. Fold-change scale was adjusted to 4; Uni $p < 0.05$ was considered statistically significant.

Figure S5: Schematic overview of the experimental design and downstream analytical methods. Abbreviations: ICH, intracerebral hemorrhage; MRI, Magnetic resonance imaging; WB, Western blotting; ELISA, Enzyme-linked immunosorbent assay; TβMCA, Tauro-β-muricholic acid; PCA, p-coumaric acid; TCA, taurocholic acid; PMA, phorbol-12-myristate-13-acetate.

Table S1. Sample sizes used in each experiment. Abbreviations: ICH, intracerebral hemorrhage; MRI, Magnetic resonance imaging; WB, Western blotting; ELISA, Enzyme-linked immunosorbent assay; TβMCA, Tauro-β-muricholic acid; PCA, p-coumaric acid; TCA, taurocholic acid; PMA, phorbol-12-myristate-13-acetate.

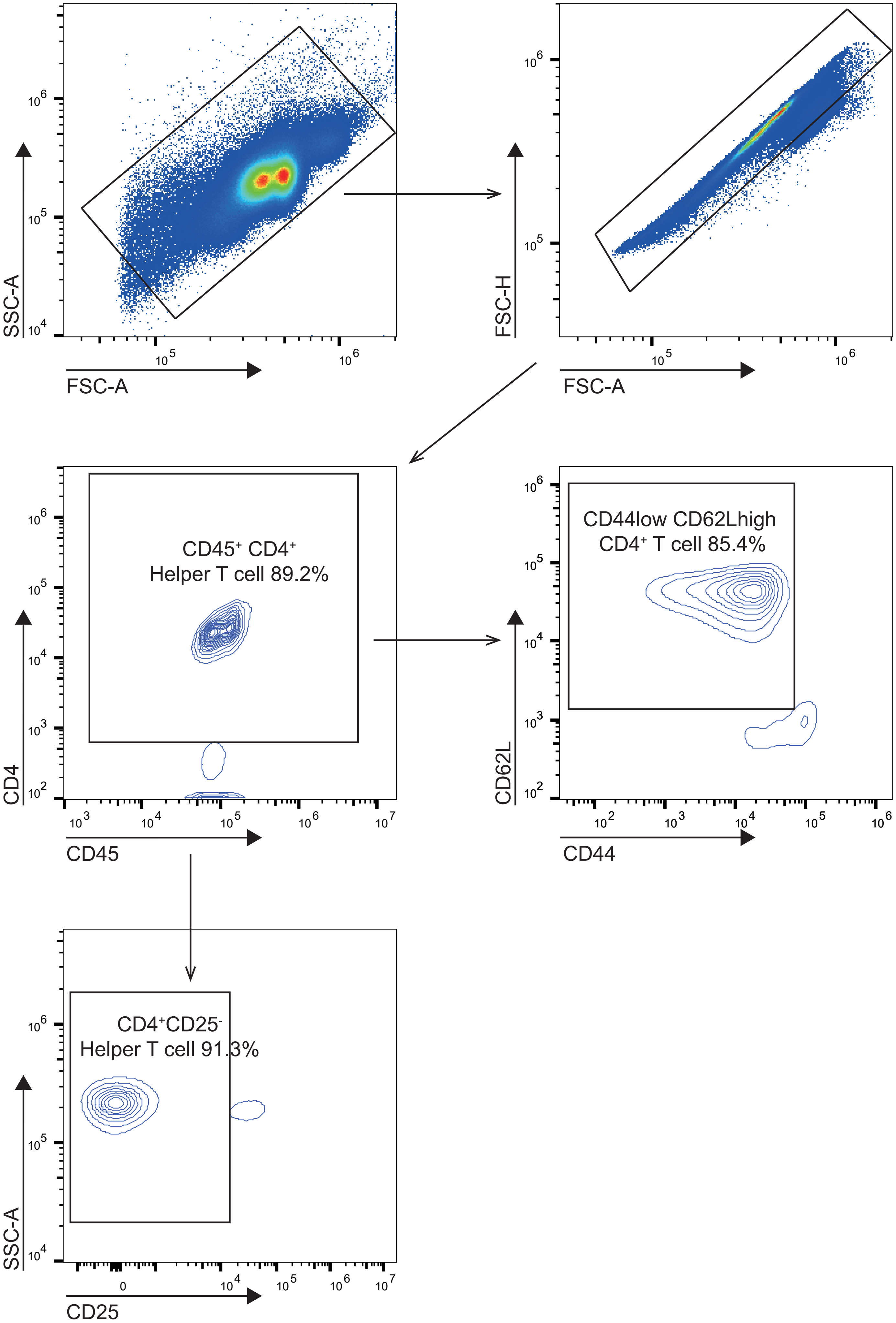
SSC-A
FSC-A
FSC-H
FSC-A
CD45⁺ CD4⁺
Helper T cell 89.2%
CD4
CD45
CD44low CD62Lhigh
CD4⁺ T cell 85.4%
CD62L
CD44
CD4⁺CD25⁻
Helper T cell 91.3%
SSC-A
CD25

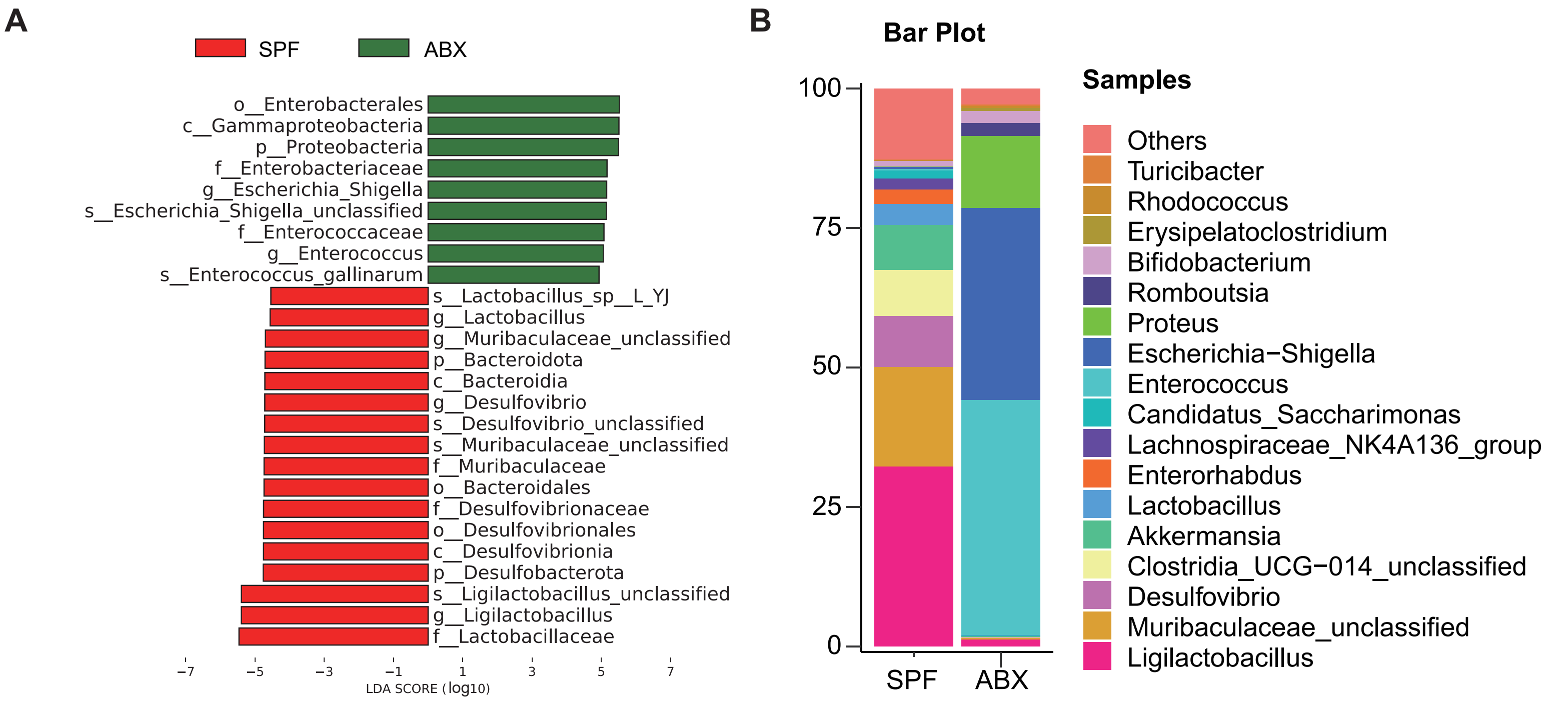
A
SPF
ABX
o__Enterobacterales
c__Gammaproteobacteria
p__Proteobacteria
f__Enterobacteriaceae
g__Escherichia_Shigella
s__Escherichia_Shigella_unclassified
f__Enterococcaceae
g__Enterococcus
s__Enterococcus_gallinarum
s__Lactobacillus_sp__L_YJ
g__Lactobacillus
g__Muribaculaceae_unclassified
p__Bacteroidota
c__Bacteroidia
g__Desulfovibrio
s__Desulfovibrio_unclassified
s__Muribaculaceae_unclassified
f__Muribaculaceae
o__Bacteroidales
f__Desulfovibrionaceae
o__Desulfovibrionales
c__Desulfovibrionia
p__Desulfobacterota
s__Ligilactobacillus_unclassified
g__Ligilactobacillus
f__Lactobacillaceae
−7 −5 −3 −1 1 3 5 7
LDA SCORE (log10)
B
Bar Plot
100
75
50
25
0
SPF
ABX
Samples
Others
Turicibacter
Rhodococcus
Erysipelatoclostridium
Bifidobacterium
Romboutsia
Proteus
Escherichia−Shigella
Enterococcus
Candidatus_Saccharimonas
Lachnospiraceae_NK4A136_group
Enterorhabdus
Lactobacillus
Akkermansia
Clostridia_UCG−014_unclassified
Desulfovibrio
Muribaculaceae_unclassified
Ligilactobacillus

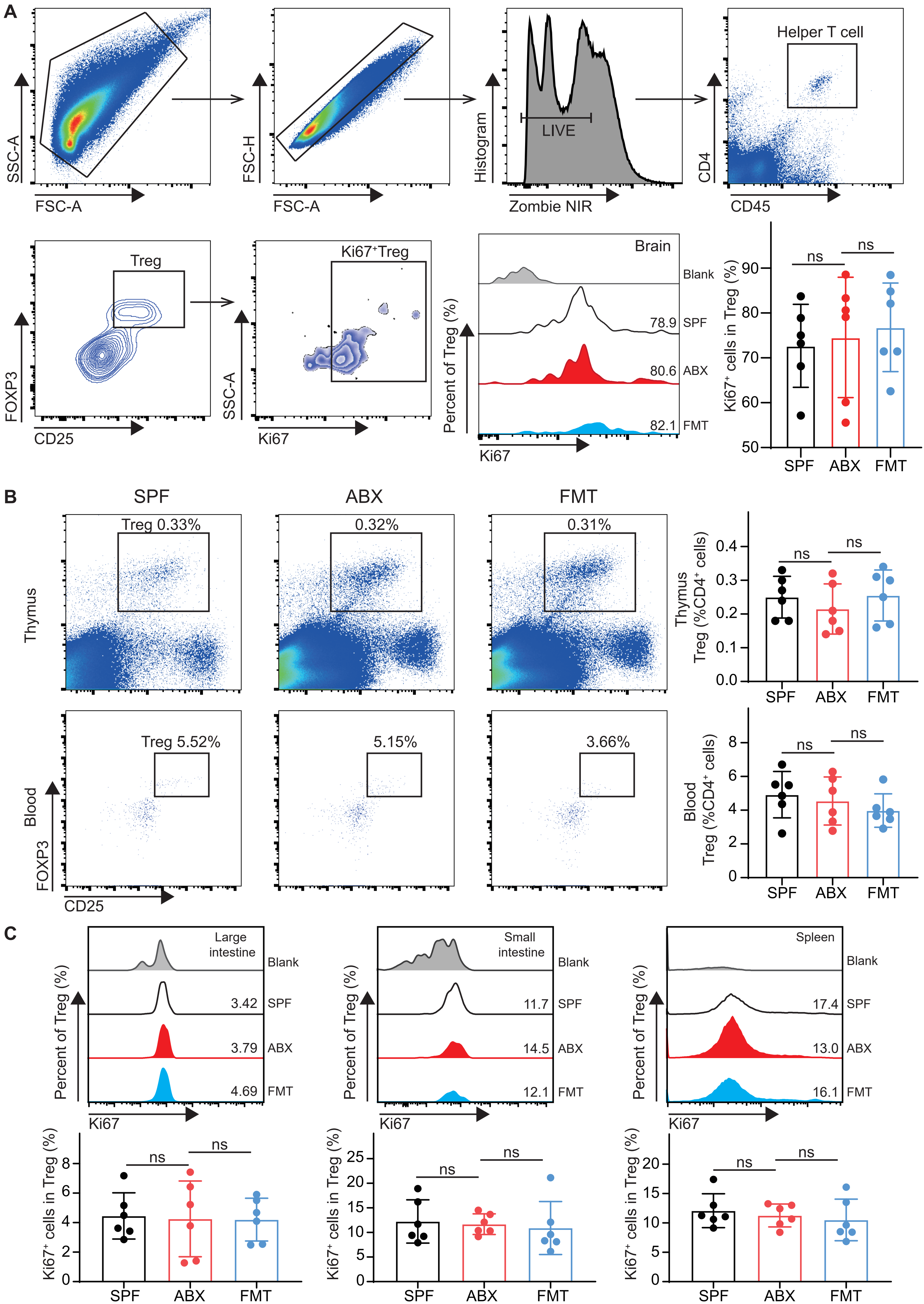
A
SSC-A
FSC-A
FSC-H
FSC-A
Histogram
LIVE
Zombie NIR
Helper T cell
CD4
CD45
Treg
FOXP3
CD25
Ki67+Treg
SSC-A
Ki67
Brain
Blank
78.9 SPF
80.6 ABX
82.1 FMT
Percent of Treg (%)
Ki67
Ki67+ cells in Treg (%)
ns
ns
SPF
ABX
FMT
B
SPF
ABX
FMT
Thymus
Treg 0.33%
0.32%
0.31%
Thymus Treg (%CD4+ cells)
Blood
Treg 5.52%
5.15%
3.66%
FOXP3
CD25
Blood Treg (%CD4+ cells)
C
Large intestine
Small intestine
Spleen
Blank
3.42 SPF
3.79 ABX
4.69 FMT
11.7 SPF
14.5 ABX
12.1 FMT
17.4 SPF
13.0 ABX
16.1 FMT
Percent of Treg (%)
Ki67
Ki67+ cells in Treg (%)

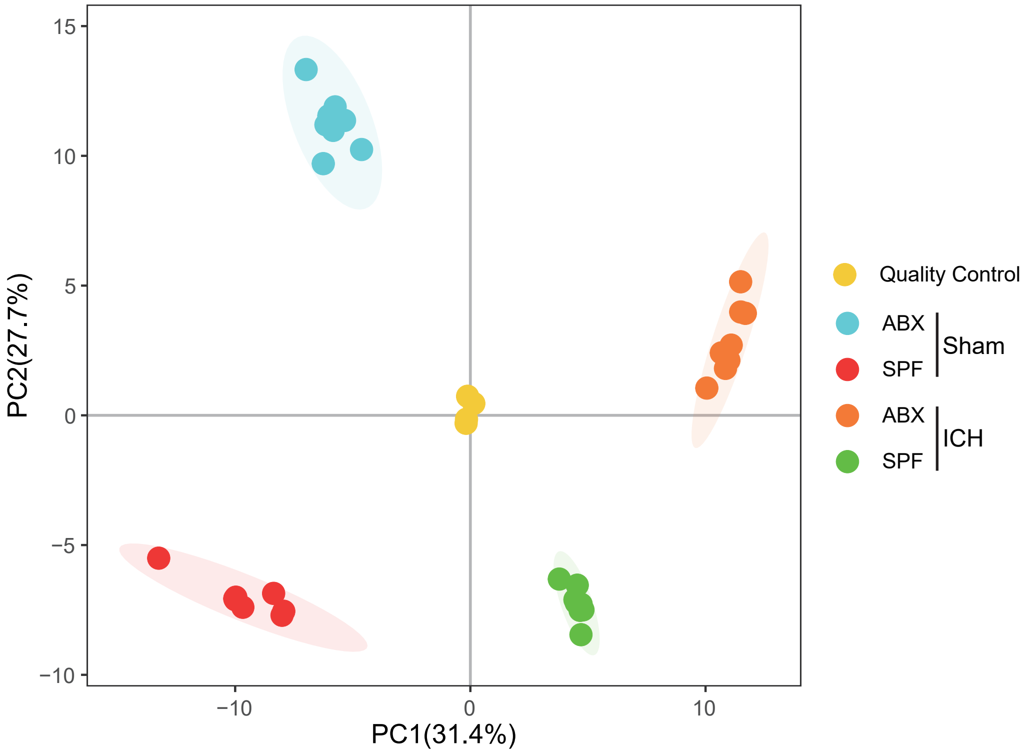

15
10
5
0
−5
−10
−10
0
10
PC2(27.7%)
PC1(31.4%)
Quality Control
ABX
SPF
Sham
ABX
SPF
ICH

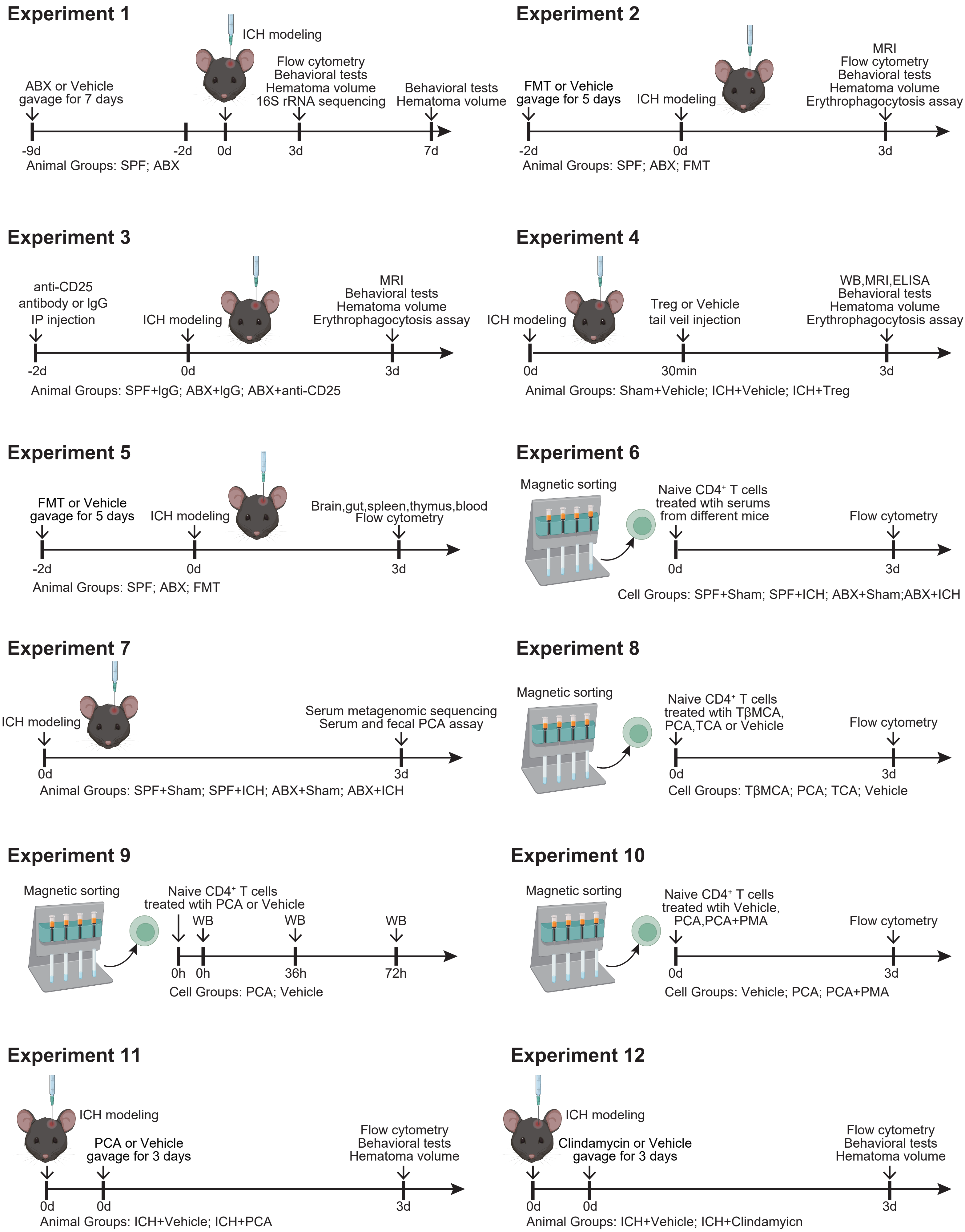
Experiment 1
ICH modeling
ABX or Vehicle
gavage for 7 days
Flow cytometry
Behavioral tests
Hematoma volume
16S rRNA sequencing
Behavioral tests
Hematoma volume
-9d
-2d
0d
3d
7d
Animal Groups: SPF; ABX
Experiment 2
FMT or Vehicle
gavage for 5 days
ICH modeling
MRI
Flow cytometry
Behavioral tests
Hematoma volume
Erythrophagocytosis assay
-2d
0d
3d
Animal Groups: SPF; ABX; FMT
Experiment 3
anti-CD25
antibody or IgG
IP injection
ICH modeling
MRI
Behavioral tests
Hematoma volume
Erythrophagocytosis assay
-2d
0d
3d
Animal Groups: SPF+IgG; ABX+IgG; ABX+anti-CD25
Experiment 4
ICH modeling
Treg or Vehicle
tail veil injection
WB,MRI,ELISA
Behavioral tests
Hematoma volume
Erythrophagocytosis assay
0d
30min
3d
Animal Groups: Sham+Vehicle; ICH+Vehicle; ICH+Treg
Experiment 5
FMT or Vehicle
gavage for 5 days
ICH modeling
Brain,gut,spleen,thymus,blood
Flow cytometry
-2d
0d
3d
Animal Groups: SPF; ABX; FMT
Experiment 6
Magnetic sorting
Naive CD4+ T cells
treated wtih serums
from different mice
Flow cytometry
0d
3d
Cell Groups: SPF+Sham; SPF+ICH; ABX+Sham;ABX+ICH
Experiment 7
ICH modeling
Serum metagenomic sequencing
Serum and fecal PCA assay
0d
3d
Animal Groups: SPF+Sham; SPF+ICH; ABX+Sham; ABX+ICH
Experiment 8
Magnetic sorting
Naive CD4+ T cells
treated wtih TβMCA,
PCA,TCA or Vehicle
Flow cytometry
0d
3d
Cell Groups: TβMCA; PCA; TCA; Vehicle
Experiment 9
Magnetic sorting
Naive CD4+ T cells
treated wtih PCA or Vehicle
WB
WB
WB
0h
0h
36h
72h
Cell Groups: PCA; Vehicle
Experiment 10
Magnetic sorting
Naive CD4+ T cells
treated wtih Vehicle,
PCA,PCA+PMA
Flow cytometry
0d
3d
Cell Groups: Vehicle; PCA; PCA+PMA
Experiment 11
ICH modeling
PCA or Vehicle
gavage for 3 days
Flow cytometry
Behavioral tests
Hematoma volume
0d
0d
3d
Animal Groups: ICH+Vehicle; ICH+PCA
Experiment 12
ICH modeling
Clindamycin or Vehicle
gavage for 3 days
Flow cytometry
Behavioral tests
Hematoma volume
0d
0d
3d
Animal Groups: ICH+Vehicle; ICH+Clindamyicn

Group	Survived	Dead	Excluded	Total	Usage
**Experiment 1**					1. 9 mice in each group in ICH 3d and ICH 7d were used for behavioral tests and hematoma volume measurement<br>2. 5 mice in each group in ICH 3d were used for 16S rRNA sequencing<br>3. 6 mice in each group in ICH 3d were used for flow cytometry
SPF	24	0	1	25	
ABX	24	0	0	24	
**Experiment 2**					1. 6 mice in each group were used for MRI<br>2. 9 mice in each group were used for flow cytometry<br>2. 9 mice in each group were used for behavioral tests and hematoma volume measurement<br>3. 6 mice in each group were used for erythrophagocytosis assay
SPF	30	1	1	32	
ABX	30	0	1	31	
FMT	30	1	2	33	
**Experiment 3**					1. 6 mice in each group were used for MRI<br>2. 9 mice in each group were used for behavioral tests and hematoma volume measurement<br>3. 6 mice in each group were used for erythrophagocytosis assay
SPF+IgG	21	1	0	22	
ABX+IgG	21	0	0	21	
ABX+anti-CD25	21	1	1	23	
**Experiment 4**					1. 6 mice in each group were used for WB<br>2. 9 mice in each group were used for MRI<br>3. 6 mice in each group were used for ELISA<br>4. 9 mice in each group were used for behavioral tests and hematoma volume measurement<br>5. 6 mice in each group were used for erythrophagocytosis assay
Sham+Vehicle	36	0	0	36	
ICH+Vehicle	36	2	2	40	
ICH+Treg	36	1	1	38	
**Experiment 5**					1. 6 mice in each group were used for brain, gut, spleen, thymus, blood Treg flow cytometry<br>2. 6 mice in each group were used for brain, gut, spleen, thymus $Ki67^+$ Treg flow cytometry
SPF	12	1	0	13	
ABX	12	0	1	13	
FMT	12	0	1	13	
**Experiment 6**					1. 6 wells of cells in each group were used for flow cytometry
SPF+Sham	6	0	0	6	
SPF+ICH	6	0	0	6	
ABX+Sham	6	0	0	6	
ABX+ICH	6	0	0	6	
**Experiment 7**					1. 8 mice in each group were used for serum metagenomic sequencing<br>2. 8 mice in SPF + Sham, SPF + ICH, and ABX + ICH group were used for serum and fecal PCA assay
SPF+Sham	16	0	0	16	
SPF+ICH	16	2	1	19	
ABX+Sham	8	0	0	8	
ABX+ICH	16	1	1	18	
**Experiment 8**					1. 6 wells of cells in each group were used for flow cytometry
TβMCA	6	0	0	6	
PCA	6	0	0	6	
TCA	6	0	0	6	
Vehicle	6	0	0	6	
**Experiment 9**					1. 6 wells of cells in each group at 0h, 36h, 72h were used for WB
Vehicle	18	0	0	18	
PCA	18	0	0	18	
**Experiment 10**					1. 6 wells of cells in each group were used for flow cytometry
Vehicle	6	0	0	6	
PCA	6	0	0	6	
PCA+PMA	6	0	0	6	
**Experiment 11**					1. 6 mice in each group were used for flow cytometry<br>2. 12 mice in each group were used for behavior tests<br>3. 9 mice in each group were used for hematoma volume measurement
ICH+Vehicle	27	2	0	29	
ICH+PCA	27	1	0	28	
**Experiment 12**					1. 6 mice in each group were used for flow cytometry<br>2. 12 mice in each group were used for behavior tests<br>3. 10 mice in each group were used for hematoma volume measurement
ICH+Vehicle	28	1	1	30	
ICH+Clindamycin	28	1	0	29	
